# Metformin as a senostatic drug enhances the anticancer efficacy of CDK4/6 inhibitor in head and neck squamous cell carcinoma

**DOI:** 10.1038/s41419-020-03126-0

**Published:** 2020-10-28

**Authors:** Qinchao Hu, Jianmin Peng, Laibo Jiang, Wuguo Li, Qiao Su, Jiayu Zhang, Huan Li, Ming Song, Bin Cheng, Juan Xia, Tong Wu

**Affiliations:** 1 Department of Oral Medicine, Hospital of Stomatology, Sun Yat-sen University, Guangzhou, China; 2 Guangdong Provincial Key Laboratory of Stomatology, Guanghua School of Stomatology, Sun Yat-sen University, Guangzhou, China; 3 Animal Experiment Center, The First Affiliated Hospital, Sun Yat-Sen University, Guangzhou, China; 4 State Key Laboratory of Oncology in South China, Collaborative Innovation Center of Cancer Medicine, Guangzhou, China; 5 Department of Intensive Care Unit, Sun Yat-sen University Cancer Center, Guangzhou, China; 6 Department of Head and Neck Surgery, Sun Yat‑sen University Cancer Center, Guangzhou, China

**Keywords:** Head and neck cancer, Cancer therapy, Head and neck cancer

## Abstract

CDK4/6 inhibitors show promising antitumor activity in a variety of solid tumors; however, their role in head and neck squamous cell carcinoma (HNSCC) requires further investigation. The senescence-associated secretory phenotype (SASP) induced by CDK4/6 inhibitors has dual effects on cancer treatment. The need to address the SASP is a serious challenge in the clinical application of CDK4/6 inhibitors. We investigated whether metformin can act as a senostatic drug to modulate the SASP and enhance the anticancer efficacy of CDK4/6 inhibitors in HNSCC. In this study, the efficacy of a combination of the CDK4/6 inhibitor LY2835219 and metformin in HNSCC was investigated in in vitro assays, an HSC6 xenograft model, and a patient-derived xenograft model. Senescence-associated β-galactosidase staining, antibody array, sphere-forming assay, and in vivo tumorigenesis assay were used to detect the impacts of metformin on the senescence and SASP induced by LY2835219. We found that LY2835219 combined with metformin synergistically inhibited HNSCC by inducing cell cycle arrest in vitro and in vivo. Metformin significantly modulated the profiles of the SASP elicited by LY2835219 by inhibiting the mTOR and stat3 pathways. The LY2835219-induced SASP resulted in upregulation of cancer stemness, while this phenomenon can be attenuated when combined with metformin. Furthermore, results showed that the stemness inhibition by metformin was associated with blockade of the IL6-stat3 axis. Survival analysis demonstrated that overexpression of IL6 and stemness markers was associated with poor survival in HNSCC patients, indicating that including metformin to target these proteins might improve patient prognosis. Collectively, our data suggest that metformin can act as a senostatic drug to enhance the anticancer efficacy of CDK4/6 inhibitors by reprogramming the profiles of the SASP.

## Introduction

Head and neck squamous cell carcinoma (HNSCC) is one of the most common cancers worldwide, accounting for ~8% of all new cancer cases in 2018^1^. Despite recent progress in treatment approaches, such as immunotherapy and targeted therapy, the 5-year survival rate of HNSCC patients is still ~60%, which is unsatisfactory^2–4^. Therefore, the development of novel therapeutic strategies for HNSCC is urgently needed.

Sustained proliferation is one of the ten hallmarks of cancer^5^. The p16-Cyclin D1-CDK4/6-Rb pathway, which regulates cell cycle progression, is frequently altered in tumors, which leads to sustained proliferation. In HNSCC, homozygous deletion or mutation of *CDKN2A* (encoding the p16 protein) is observed in 58% of patients, and amplification of *CCND1* (encoding the Cyclin D1 protein) is observed in 31% of patients^6^. This suggests that targeting the p16-Cyclin D1-CDK4/6-Rb cell cycle pathway may effectively inhibit HNSCC. Currently, three CDK4/6 inhibitors including PD0332991 (palbociclib), LEE011 (ribociclib), and LY2835219 (abemaciclib) are approved by the food and drug administration (FDA) for clinical treatment of advanced metastatic breast cancer. CDK4/6 inhibitors also show promising antitumor activity in a variety of solid tumors^7,8^. However, the antitumor effect of CDK4/6 inhibitors on HNSCC needs to be confirmed, and the underlying mechanism requires further investigation.

It is widely recognized that CDK4/6 inhibitors exert their antitumor effects by inducing cell cycle arrest, which is closely linked with cell senescence. Therefore, therapy-induced senescence (TIS) was found to be a common outcome after CDK4/6 inhibitor treatment^9^. Senescent cells lose the ability to proliferate but remain viable and metabolically active. A variety of bioactive molecules, including proinflammatory cytokines, chemokines, growth factors, and proteases, are secreted by senescent cells, which is termed the senescence-associated secretory phenotype (SASP). Accumulating evidence suggests that the SASP is a “double-edged sword” in cancer treatment that can either inhibit or promote cancer^10,11^. Cytokines such as IL1α and TGFβ can induce paracrine senescence in neighboring cancer cells to enhance growth suppression^12,13^. However, cytokines such as IL6, IL8, CXCL1, and VEGF can accelerate tumor progression by promoting tumor cell proliferation, migration, invasion, angiogenesis, and stemness-related factor upregulation^14–20^. Moreover, CCL2/MCP1 recruit myeloid-derived suppressor cells to the tumor site to alter the microenvironment and promote tumor progression^21,22^. Therefore, understanding how the profiles of the SASP can be modulated to favor positive treatment outcomes may be the key to improving the anticancer efficacy of CDK4/6 inhibitors.

Given the dual nature of the SASP in cancer treatment, a two-step senescence-focused cancer therapy concept was proposed^23^. Senescence-inducing chemotherapy followed by senotherapy is used to eliminate the adverse effects of the SASP and enhance antitumor efficacy. Senotherapy agents can be classified into two types: senolytics and senostatics. Senolytics are small-molecule agents that selectively kill senescent cells so that the sources of the SASP are eliminated. In contrast to senolytics, senostatics do not aim to kill senescent cells but instead suppress or regulate the SASP. Senostatics are now showing significant promise as an adjuvant tumor therapy^24^. Metformin, a widely used hypoglycemic drug, was reported to regulate the SASP in senescent fibroblasts, suggesting that metformin has the potential to act as a senostatic drug^25^. In addition, our previous study found that metformin exhibited anticancer activity in HNSCC, and it has been indicated to be an adjuvant agent that can enhance conventional cancer treatment^26,27^. This evidence strongly indicates that metformin may enhance the anticancer efficacy of CDK4/6 inhibitors in HNSCC by modulating the therapy-induced SASP.

In this study, we found that the CDK4/6 inhibitor LY2835219 combined with metformin synergistically inhibited HNSCC by inducing cell cycle arrest in vitro and in vivo. Furthermore, metformin enhanced anticancer efficacy by acting as a senostatic drug to modulate the profiles of the CDK4/6 inhibitor-induced SASP and thus blocked SASP-induced stemness.

## Materials and methods

### Cell lines and cell cultures

The human HNSCC cell line Cal27 was purchased from ATCC (Rockville, MD, USA), and the cell lines HSC3 and HSC6 were provided by J. Silvio Gutkind (NIH, Bethesda, MD, USA). The human breast cancer cell line MCF7 was purchased from ATCC. The cell lines used in this study were authenticated using short tandem repeat (STR) analysis and regularly tested for mycoplasma. Cells were cultured in Dulbecco’s Modified Eagle Medium (DMEM) supplemented with 10% fetal bovine serum and 1% penicillin/streptomycin at 37 °C in a 5% CO_2_ atmosphere.

### Chemical reagents and antibodies

LY2835219 was purchased from MedChemExpress (Monmouth Junction, NJ, USA). Metformin was obtained from Sigma-Aldrich (St. Louis, MO, USA). S3I-201 and INK-128 were obtained from Selleck Chemicals (Houston, TX, USA). Recombinant human IL6 was obtained from Novoprotein (Shanghai, China). An anti-IL6 neutralizing antibody was purchased from Sino Biological (Beijing, China). Antibodies against p21 (#2947), p-Rb (Ser780, #8180), Rb (#9309), p-mTOR (Ser2448, #5536), mTOR (#2983), p-stat3 (Tyr705, #9145), stat3 (#9139), CD44 (#3570), Nanog (#3580), and ALDH1A1 (#54135) were purchased from Cell Signaling Technology (Beverly, MA, USA). An antibody against p-Rb (Ser780, #AF3103) for immunohistochemistry (IHC) was obtained from Affinity Biosciences (Cincinnati, OH, USA). An antibody against p16 (#377412) was purchased from Santa Cruz Biotechnology (Dallas, TX, USA). Antibodies against Ki67 (#arg53222) and PCNA (#arg62605) were obtained from Arigo Biolaboratories (Taiwan, China). Antibodies against IL6 (#ab6672), CD11b (#ab133357), and NKp46 (#ab233558) were purchased from Abcam (Cambridge, UK). An antibody against Gr1 (#GB11229) was purchased from Servicebio (Wuhan, China). An Alexa Fluor® 700-conjugated anti-mouse/human CD11b antibody (#101222) was purchased from BioLegend (San Diego, CA, USA). An APC-conjugated anti-mouse/human Arg1 antibody (#17-3697-80) was purchased from eBioscience (San Diego, CA, USA).

### Cell viability assay and synergy analysis

Cells were seeded in 96-well plates, allowed to adhere overnight, and then treated with the indicated compounds for 72 h. Cell viability was determined using a Cell Counting Kit (Dojindo, Kumamoto, Japan) according to the manufacturer’s instructions. Synergy analysis was performed using the Chou–Talalay method and Compusyn software. The interaction was determined based on the combination index (CI): synergism (CI < 1), additive effect (CI = 1), or antagonism (CI > 1).

### Colony formation assay

Cells were seeded in 6-well plates, allowed to adhere overnight, and then treated with the indicated compounds. The medium was changed twice a week with fresh reagents added. After 10–14 days, the cells were fixed in 4% formaldehyde and stained with crystal violet. Colonies (>50 cells/clone) were counted using ImageJ (NIH, Bethesda, MD, USA).

### Cell cycle analysis

Cells were treated with the indicated compounds for 24 h, harvested and then stained with a cell cycle staining kit (Lianke, China) according to the manufacturer’s instructions. The cell cycle distribution was analyzed by flow cytometry (Beckman Coulter, California, USA).

### Western blot (WB) analysis

Cells were collected and lysed in RIPA buffer (Sigma-Aldrich) supplemented with protease and phosphatase inhibitors. Equal amounts of protein were loaded onto an 8 or 10% SDS-PAGE gel for separation and then transferred to a PVDF membrane (Millipore, MA, USA). PVDF membranes were blocked with milk and incubated with primary antibodies (antibodies were specific for p16, p21, pRb, Rb, p-mTOR, mTOR, p-stat3, stat3, ALDH1A1, CD44, Nanog, or GAPDH), followed by incubation with a secondary antibody. The signal was visualized using an enhanced chemiluminescence (ECL) detection system (Millipore).

### Immunostaining

OCT-embedded frozen tumor tissues derived from a PDX model were used for immunofluorescence staining. In brief, 8-μm-thick cryosections were prepared and fixed in ice-cold acetone for 10 min. After washing with PBS, the sections were blocked with 10% normal goat serum for 1 h at room temperature and then incubated with primary antibodies overnight at 4 °C. The sections were subsequently washed with PBST and then incubated with secondary antibodies for 1 h at room temperature. Nuclear counterstaining was performed using 4′,6-diamidino-2-phenylindole (DAPI).

Paraffin-embedded tumor tissues derived from an HSC6 xenograft model were evaluated by IHC as previously described^28^.

Cells treated with conditioned medium (CM) from different groups were prepared for immunofluorescence analysis. After treatment, cells were fixed with 4% formaldehyde for 10 min, permeabilized with 0.1% Triton X-100 for 15 min, and blocked with 5% bovine serum albumin (BSA) for 1 h at room temperature. Next, the cells were incubated with primary antibodies overnight at 4 °C and subsequently incubated with secondary antibodies for 1 h at room temperature. DAPI was used to counterstain the nuclei. Images were obtained with a laser scanning confocal microscope (Carl Zeiss AG, Germany).

### Senescence-associated β-galactosidase (SA-β-gal) staining

Cells were treated with the indicated reagents for 48 h before analysis using an SA-β-gal staining kit (Beyotime, China) according to the manufacturer’s protocol.

### Quantitative RT-PCR analyses

Total RNA was extracted by TRIzol (Invitrogen, Carlsbad, CA, USA), and cDNA was synthesized using the Transcriptor First Strand cDNA Synthesis Kit (Roche, Mannheim, Germany). qRT-PCR was performed with SYBR Green I Master Mix (Roche) on a Light Cycler 480 system (Roche). The PCR primers are listed in Supplementary Table 1. All results were normalized to those for GAPDH.

### Antibody array

To analyze cytokines in CM from different groups, the human cytokine antibody array AAH-CYT-G1000 (RayBiotech, Norcross, GA, USA) was adopted. In brief, Cal27 cells were treated with different reagents (1.25 μM LY2835219, 10 mM metformin, or a combination) for 48 h. Then, the cells were washed with PBS and incubated in fresh medium for 24 h to generate CM. The CM was then obtained, centrifuged, and normalized by cell count. The normalized CM was subsequently applied to the antibody arrays as recommended by the supplier.

### Sphere-forming assay

Cells were treated with CM from different groups for 48 h and then plated in ultralow-attachment 24-well plates (Corning, Steuben, NY, USA) at a density of 1000 cells/well. The cells were cultured in serum-free DMEM/F12 medium supplemented with 20 ng/ml human recombinant EGF, 10 ng/ml human recombinant bFGF, and B27 (Invitrogen, Carlsbad, CA, USA). After 14 days, the number of spheres (diameter > 50 µm) was counted.

### Flow cytometry analysis

Flow cytometry analysis was performed to detect the expression of the stemness marker ALDH1A1 after cells were treated with CM. In brief, cells were fixed with 4% formaldehyde for 15 min at room temperature and permeabilized with 90% ice-cold methanol for 30 min at 4 °C. Subsequently, the cells were incubated with an anti-ALDH1A1 primary antibody for 1 h and Alexa Fluor 594-conjugated secondary antibody for 30 min at room temperature. Then, the cells were analyzed on a flow cytometer.

### ELISA assay

The normalized CM described above was also analyzed by ELISA. A human IL6 ELISA kit was purchased from Boster (Wuhan, China). The assay was performed following the procedures described by the manufacturer.

### Animal studies

For an HSC6 xenograft model, 5 × 10^6^ HSC6 cells were injected subcutaneously into the right armpit of nude mice. When the tumors reached a mean size of ~100 mm^3^, the mice were randomized into four groups (8 mice per group) and treated as follows: vehicle control, LY2835219 (25 mg/kg/d, p.o., qd), metformin (100 mg/kg/d, i.p., qd), and LY2835219 + metformin (combination). LY2835219 was dissolved in 1% HEC in 20 mM phosphate buffer (pH 2.0) and administered orally by gavage (final volume of 0.1 mL) at the indicated dose and schedule. Metformin was dissolved in PBS and administered by intraperitoneal injection. Mouse body weights and tumor sizes were recorded every 2 days. Tumor volume (V) was estimated according to the following formula: *V* = (length × width^2^)/2. The mice were sacrificed 3 weeks after treatment. The tumor tissues were removed, weighed and then fixed in 4% formaldehyde for paraffin embedding.

For a patient-derived xenograft (PDX) model, tumor samples were collected at the Department of Head and Neck Surgery, Sun Yat-sen University Cancer Center. Prior informed consent was obtained for all collections. In brief, freshly resected tumors were intensively washed and cut into small pieces (diameter, 0.8–1.5 mm) in antibiotic-containing DMEM. Then, the tumor pieces were implanted subcutaneously into the flanks of nude mice (P0 xenografts). When the tumor size reached 1500 mm^3^, the tumors were dissected, processed and reinjected for expansion (P1 xenografts). This process was further repeated, and the animal study was performed with P2 xenografts. Histopathological HE staining indicated that the histological characteristics of the PDX tumors after passaging were similar to those of the primary tumor (Supplementary Fig. S1). When the xenografts reached a mean size of ~100 mm^3^, the mice were randomized into four groups (8 mice per group) and treated as follows: vehicle control, LY2835219 (40 mg/kg/d, p.o., qd), metformin (200 mg/kg/d, i.p., qd), and LY2835219 + metformin (combination). The mice were sacrificed 3 weeks after treatment. Tumor tissues were removed, weighed and then embedded in OCT.

To detect the tumor formation ability of cells pretreated with CM, Cal27 cells were incubated with CM from different groups for 96 h. Then, relatively lower concentration of cells (2 × 10^5^ cells per mouse) in different groups were injected subcutaneously into the right armpit of nude mice (8 mice per group) in a blinded fashion. After 1 month, the mice were sacrificed. The numbers of tumors formed were recorded. The tumor tissues were removed and then embedded in OCT.

All of the animal procedures were conducted in accordance with the Guidelines for the Care and Use of Laboratory Animals and were approved by the Institutional Animal Care and Use Committee at Sun Yat-sen University.

### The Cancer Genome Atlas (TCGA) database analysis

mRNA sequencing data and clinical information were downloaded from the TCGA data portal. A total of 520 HNSCC cases with both sequencing data and corresponding clinical information were obtained.

### Statistical analysis

Data are presented as the mean ± SD. Comparisons between two groups were performed using Student’s *t*-test. When comparing data from multiple groups, one-way ANOVA (no matching) was used with the Tukey procedure to adjust for multiple comparisons. Sample sizes and replicates were chosen according to previous studies and are indicated in each method described above. Survival curves were plotted by the Kaplan–Meier method and compared by the log-rank test. Statistical analysis was performed with GraphPad Prism 5.0 (GraphPad software, San Diego, CA, USA) and SPSS 23.0 software (IBM, Armonk, NY, USA). A two-tailed *P* value < 0.05 was considered statistically significant.

## Results

### Combination of a CDK4/6 inhibitor with metformin shows synergistic effects on HNSCC in vitro and in vivo

**Fig. 1:**
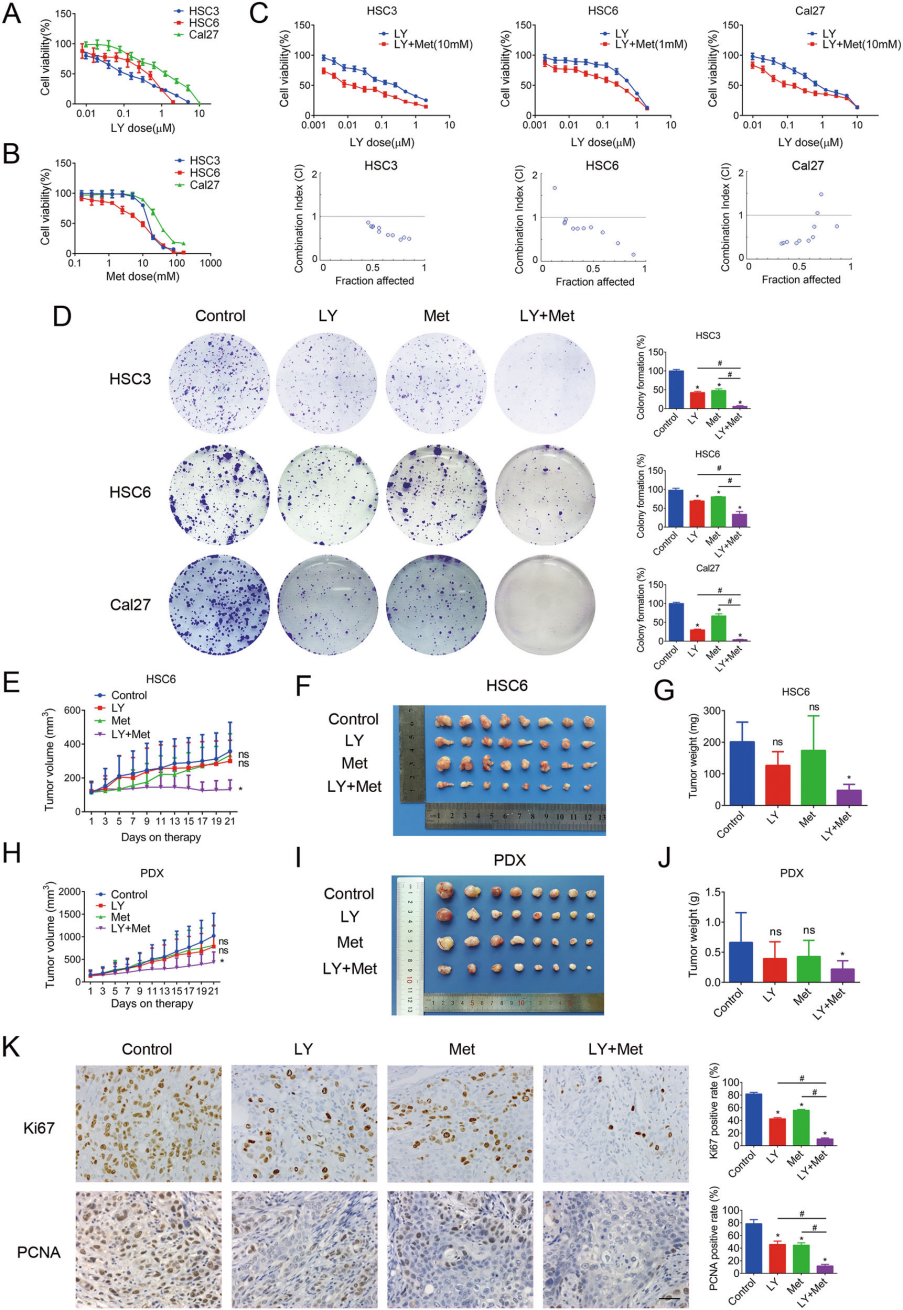
Combining a CDK4/6 inhibitor and metformin synergistically inhibited HNSCC in vitro and in vivo. **A** A CCK8 assay showed that the CDK4/6 inhibitor LY2835219 reduced the cell viability of the HNSCC cell lines HSC3, HSC6, and Cal27. **B** A CCK8 assay showed that metformin reduced the cell viability of HSC3, HSC6, and Cal27 cells. **C** Cells were treated with increasing concentrations of LY2835219 with or without metformin. According to the CI values calculated by Compusyn, the combination of LY2835219 and metformin exhibited a synergistic effect (CI < 1). **D** A clonogenic assay indicated that LY2835219 and metformin synergistically inhibited the colony-forming ability of HNSCC cell lines. **E**–**G** LY2835219 (25 mg/kg/d, p.o., qd) and metformin (100 mg/kg/d, i.p., qd) synergistically inhibited tumor growth in an HSC6 xenograft model. **H**–**J** LY2835219 (40 mg/kg/d, p.o., qd) and metformin (200 mg/kg/d, i.p., qd) synergistically inhibited tumor growth in a PDX model. **K** Immunohistochemistry results (400×) showed that LY2835219 and metformin synergistically inhibited the expression of Ki67 and PCNA in HSC6 xenograft tumors. Bar: 100 μm. *P < 0.05 when compared with the control group; ^#^*P* < 0.05 when compared with the combined group; ns indicates no significant difference; one-way ANOVA. CI: combination index; LY: LY2835219; Met: Metformin; PDX, Patient-derived xenograft.

To reveal the expression pattern of components of the CDK4/CDK6/cyclin D1 pathway in HNSCC, a tumor cohort from the TCGA database was analyzed. Consistent with the previous studies^6,29^, our results confirmed that *CCND1*, *CDK4*, and *CDK6* were all overexpressed in HNSCC (Supplementary Fig. S2A). In addition, patients with high expression of these genes exhibited poor survival^30^ (Supplementary Fig. S2B). These data suggest the potential of CDK4/6 inhibitors in HNSCC. In an in vitro study, three HNSCC cell lines (HSC3, HSC6, and Cal27) were treated with LY2835219, a CDK4/6 inhibitor. A CCK8 assay revealed that LY2835219 significantly reduced cell viability in a dose-dependent manner (Fig. 1A). The IC_50_ values of LY2835219 were 0.10, 0.30, and 1.25 μM for HSC3, HSC6, and Cal27 cells, respectively. Similarly, metformin treatment significantly inhibited the growth of cells (Fig. 1B). The IC_50_ values of metformin were 18.32, 6.50, and 39.48 mM for HSC3, HSC6, and Cal27 cells, respectively. Then, we tested whether combining metformin and LY2835219 has a synergistic effect on cell viability. Cells were treated with increasing concentrations of LY2835219 with or without metformin (the concentration of metformin was IC_10_–IC_20_). The combined groups exhibited more potent inhibitory effects on cell growth than the single-treatment groups (Fig. 1C). According to the CI values, the combination therapy exhibited a synergistic effect (CI < 1) that reduced cell viability (Fig. 1C). Furthermore, a clonogenic assay indicated that LY2835219 and metformin synergistically inhibited the colony formation ability of HNSCC cells (Fig. 1D).

Next, we evaluated the efficacy of the LY2835219 and metformin combination in an HSC6 xenograft model established with nude mice. To illustrate the synergistic effect, relatively low concentrations of LY2835219 (25 mg/kg/d) and metformin (100 mg/kg/d) were selected, according to previous studies^31–36^. Mice bearing tumor xenografts were treated with vehicle, LY2835219 only, metformin only, or a combination of LY2835219 and metformin. While each individual drug failed to yield significant therapeutic responses, the combined treatment significantly inhibited tumor growth (Fig. 1E, F). The mean weights of tumors excised from mice were 201.3 ± 62.51, 126.5 ± 43.59, 173.9 ± 109.8, and 47.5 ± 19.03 mg for the control, LY2835219, metformin, and combination groups (*P* < 0.05, compared with the control, Fig. 1G), respectively. To further assess the clinical therapeutic potential of the CDK4/6 inhibitor and metformin combination, we tested its efficacy using a primary HNSCC PDX model. Mice bearing PDX tumors were treated with vehicle, LY2835219 only (40 mg/kg/d), metformin only (200 mg/kg/d), or a combination of LY2835219 + metformin. While none of the individual drugs yielded significant suppression, the combined treatment significantly suppressed tumor growth (Fig. 1H, I). The combination therapy significantly decreased tumor weight in the PDX model (Fig. 1J). In addition, mouse body weight was not affected by individual or combined treatments (Supplementary Fig. S3A). No overt changes in the pathology of the liver or kidney were noted in any of the treatment groups (Supplementary Fig. S3B). IHC results showed that the monotherapies slightly decreased the positive rates of Ki67 and PCNA in tumors, while the combination treatment exerted a remarkable effect (Fig. 1K). Taken together, these results indicated that the combination of LY2835219 and metformin exhibited synergistic effects on HNSCC in vitro and in vivo.

### CDK4/6 inhibitor in combination with metformin significantly promotes cell cycle arrest

**Fig. 2:**
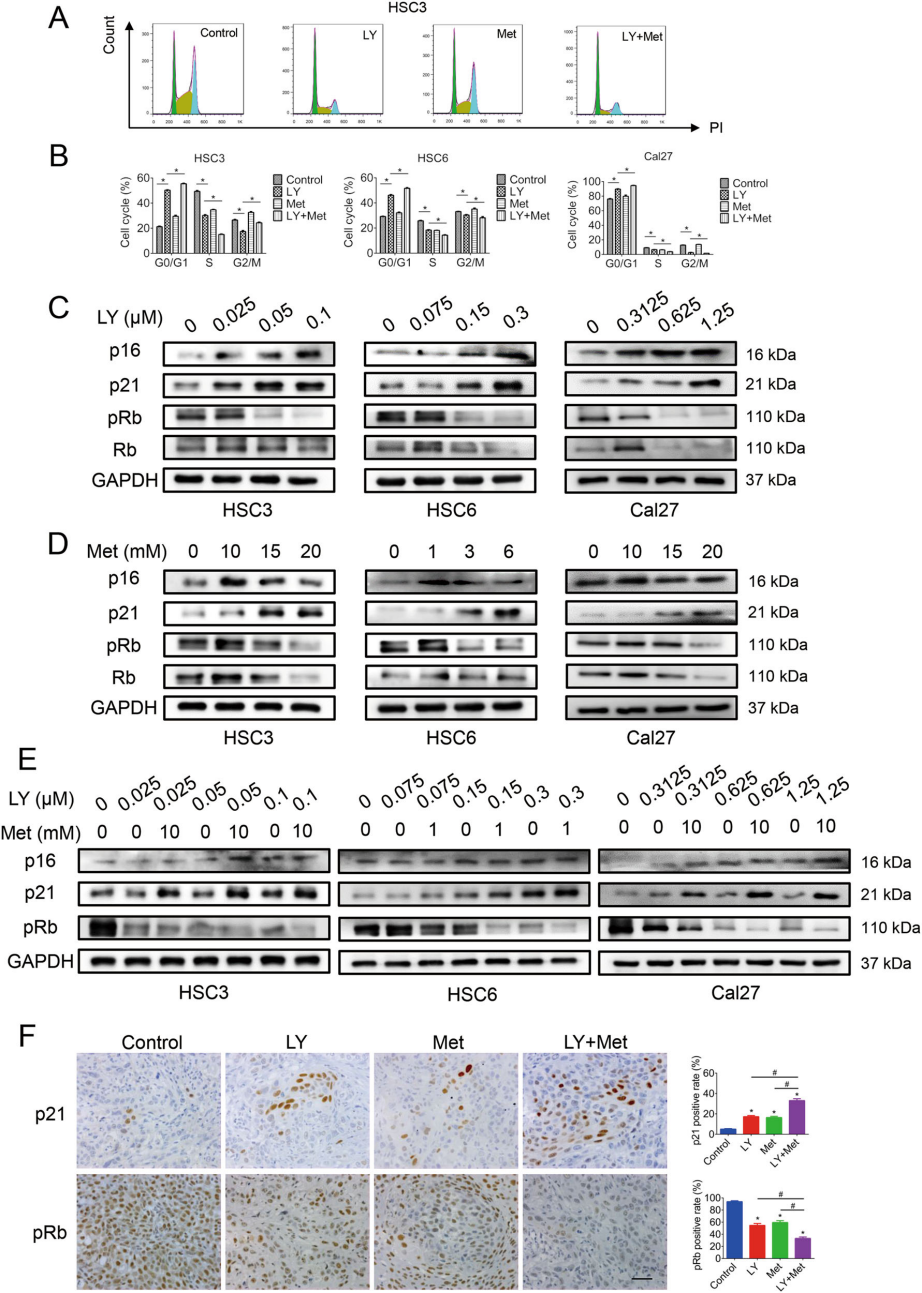
CDK4/6 inhibitor combined with metformin promoted cell cycle arrest. **A**, **B** Flow cytometry results showed that the combination of the CDK4/6 inhibitor LY2835219 (HSC3: 0.1 μM; HSC6: 0.3 μM; Cal27: 1.25 μM) with metformin (HSC3: 10 mM; HSC6: 1 mM; Cal27: 10 mM) induced cell cycle arrest in the G0/G1 phase. **C** Western blot results showed that LY2835219 upregulated the expression of p16 and p21 and downregulated the expression of pRb. **D** Western blot results showed that metformin upregulated the expression of p16 and p21 and downregulated the expression of pRb. **E** Compared with LY2835219 monotherapy, the combination treatment significantly upregulated p21 expression and suppressed p-Rb expression. **F** Immunohistochemistry results (400×) showed that LY2835219 and metformin synergistically upregulated p21 expression and downregulated pRb expression in HSC6 xenograft tumors. Bar: 100 μm. *P < 0.05 when compared with the control group; ^#^*P* < 0.05 when compared with the combined group; one-way ANOVA. LY: LY2835219; Met: Metformin.

To examine the mechanisms underlying the inhibition of tumor growth by the combination treatment, we analyzed alterations in cell cycle progression. After treatment with LY2835219 for 24 h, the cell cycle was distinctly arrested in the G0/G1 phase, accompanied by decreased proportions of cells in the S and G2/M phases (Fig. 2A, B). Metformin elicited slight cell cycle arrest in the G0/G1 and G2/M phases and decreased the proportion of cells in the S phase. The combined treatment resulted in more efficient arrest in the G0/G1 phase and a decrease in the proportion of cells in the S phase (Fig. 2A, B). Next, we detected the expression of G1 checkpoint proteins. The levels of p16, the main protein regulating early G1 phase progression, and p21, the main protein regulating late G1 phase progression, were upregulated by LY2835219 in a dose-dependent manner (Fig. 2C). Metformin also upregulated p21 expression, but its effect on p16 expression was relatively mild (Fig. 2D). Both agents reduced Rb phosphorylation (Fig. 2C, D). Compared with LY2835219 monotherapy, the combination treatment significantly upregulated p21 expression and suppressed p-Rb expression, while the alteration in p16 expression was not as prominent (Fig. 2E). Furthermore, p21 upregulation and p-RB downregulation were confirmed by IHC in xenograft tumor tissues (Fig. 2F). These results suggested that the combination therapy might synergistically induce cell cycle arrest through the p21-pRb pathway.

Considering that cell apoptosis is also an important mechanism of tumor suppression, we detected cell apoptosis after treatment. However, the results showed that little cell apoptosis was elicited by LY2835219, and combination with metformin did not elevate the apoptotic cell proportion either (Supplementary Fig. S4). These observations indicated that cell apoptosis might not be the main mechanism by which the combination therapy exerts its effect.

### Metformin modulates the profiles of the SASP induced by the CDK4/6 inhibitor by inhibiting the mTOR and stat3 pathways

**Fig. 3:**
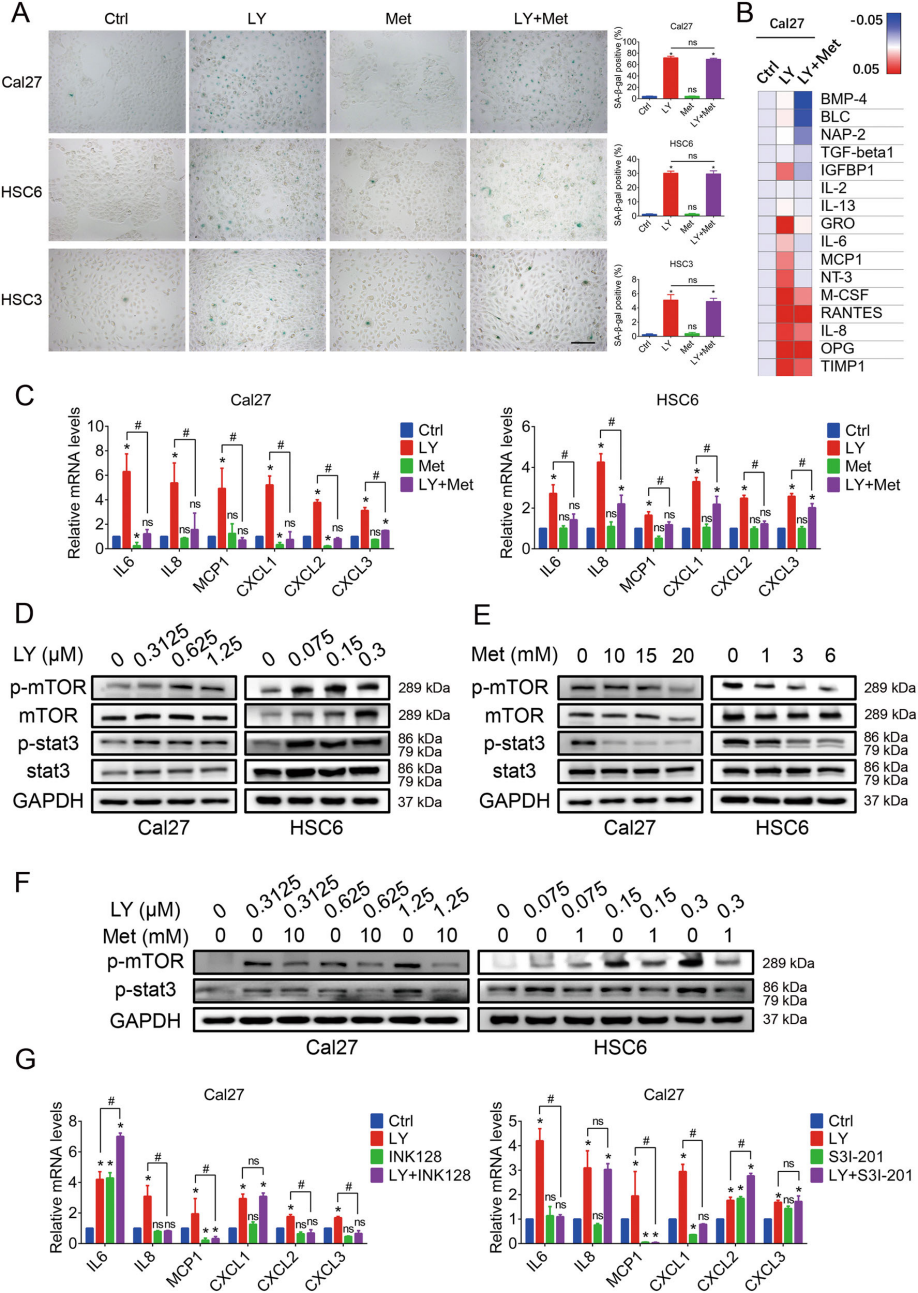
Metformin modulated the profiles of the SASP induced by a CDK4/6 inhibitor by inhibiting the mTOR and stat3 pathways. **A** SA-β-gal staining (100×) showed that remarkable senescence in cells was elicited by the CDK4/6 inhibitor LY2835219 (Cal27: 1.25 μM; HSC6: 0.3 μM; HSC3: 0.1 μM) but not by metformin (Cal27: 10 mM; HSC6: 1 mM; HSC3: 10 mM). Compared with LY2835219 monotherapy, the combination including metformin had no significant impact on the proportion of senescent cells. Bar: 400 μm. **B** Antibody array results indicated that the levels of a series of tumor-promoting SASP factors (such as IL6, IL8, MCP1, and GRO) were upregulated by LY2835219, while the combination including metformin inhibited this upregulation. **C** qRT-PCR results confirmed the modulation of the SASP by metformin. **D** Western blot results showed that the mTOR and stat3 pathways were activated by LY2835219. **E** Metformin treatment inhibited the mTOR and stat3 pathways. **F** The combination treatment inhibited the stat3 and mTOR pathways, which were activated by LY2835219 monotherapy. **G** qRT-PCR results confirmed that both INK-128 (a selective mTOR inhibitor) and S3I-201 (a selective stat3 inhibitor) could suppress the upregulation of the expression of some of the SASP factors induced by LY2835219. *P < 0.05 when compared with the control group; ^#^*P* < 0.05 when compared with the combined group; ns indicates no significant difference; one-way ANOVA. LY: LY2835219; Met: metformin.

After inducing cell cycle arrest, CDK4/6 inhibitors always make the cells into a state of senescence. Thus, we analyzed cell senescence after treatment. As shown in Fig. 3A, obvious SA-β-gal-positive cells were observed after LY2835219 treatment, while the positive rate of senescent cells did not significantly change after metformin treatment. Compared with LY2835219 monotherapy, the combination therapy did not alter the proportion of senescent cells. However, we found that the profiles of the SASP, an important characteristic of senescent cells, were reprogrammed after combination with metformin. The CM of cells in each group was collected after treatment for analysis using antibody arrays. The results showed that several cytokines reported to play important roles in tumor promotion, such as IL6, IL8, MCP1, and GRO (CXCL1, 2, 3), exhibited upregulated expression in the LY2835219 monotherapy group and downregulated expression in the combination therapy group (Fig. 3B). This observation was also confirmed by qPCR (Fig. 3C). Then, we investigated whether this effect also exists in other cancer types. As CDK4/6 inhibitors are approved for the clinical treatment of breast cancer, the MCF7 cell line was treated with LY2835219 and metformin for confirmation. The results consistently showed that metformin inhibited the upregulation of the levels of the above-mentioned cytokines by LY2835219 in MCF7 cells (Supplementary Fig. S5). Interestingly, several other cytokines reported to be associated with senescence maintenance and tumor-suppression, such as IL1α, IL1β, TGF-β, and CCL5, exhibited upregulated expression in the LY2835219 monotherapy group but did not exhibit downregulated expression in the combination group (Supplementary Fig. S6A). Moreover, the CDK4/6 inhibitor-induced SASP was able to promote and maintain cell senescence, which resulted in proliferation inhibition, and these effects were not repressed in the combination CM group (Supplementary Fig. S6B–D).

To determine the underlying mechanism, WB analysis was performed. LY2835219 treatment significantly activated the mTOR and stat3 pathways, while the combination with metformin suppressed the activated mTOR and stat3 pathways (Fig. 3D–F). These results indicated that the combination treatment might decrease the levels of the tumor-promoting cytokines mentioned above by inhibiting the mTOR and stat3 pathways. For confirmation, two specific inhibitors, INK-128 (a selective mTOR inhibitor) and S3I-201 (a selective stat3 inhibitor), were applied. qPCR results showed that both INK-128 and S3I-201 could suppress some of the cytokines involved (Fig. 3G), suggesting that metformin inhibits both the mTOR pathway and the stat3 pathway to regulate these tumor-promoting cytokines. Taken together, our results revealed that metformin modulated the profiles of the SASP induced by LY2835219 by inhibiting the mTOR and stat3 pathways.

### Metformin blocks the SASP-induced stemness caused by a CDK4/6 inhibitor

**Fig. 4:**
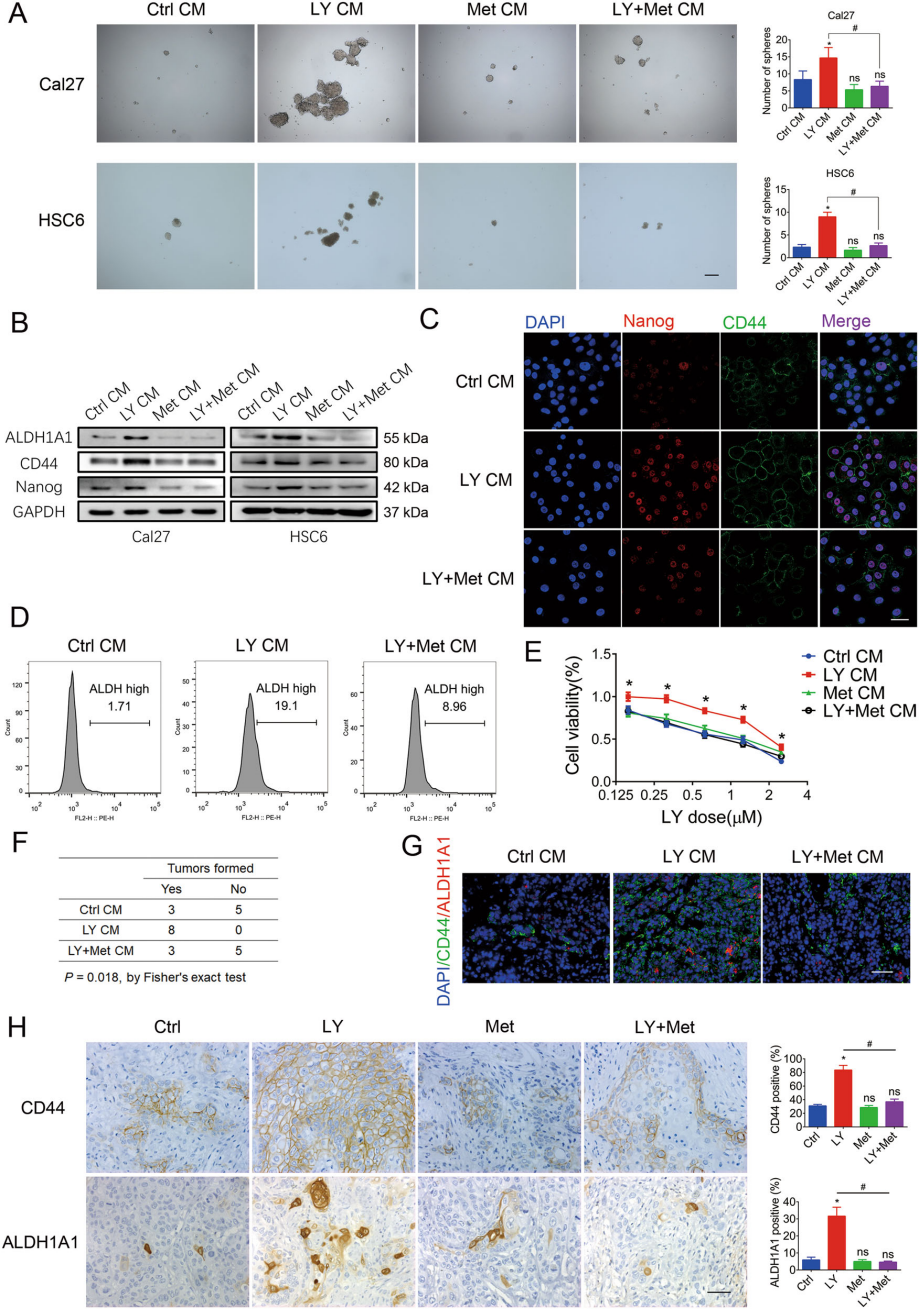
Combination of LY2835219 with metformin blocked the stemness induced by the SASP. **A** A sphere-forming assay showed that LY CM significantly enhanced the sphere-forming ability, while LY + Met CM attenuated this effect. Bar: 500 μm. Western blot (**B**), laser confocal microscopy (**C**), and flow cytometry (**D**) results showed that the expression of the cancer stem cell markers ALDH1A1, CD44, and Nanog was upregulated in cells stimulated with LY CM, but this upregulation was diminished when cells were treated with LY + Met CM. Bar: 50 μm. **E** Cells pre-stimulated with CM from different groups were then treated with LY2835219. A CCK8 assay revealed that the cells stimulated with LY CM had a significantly lower inhibition ratio than the control cells, while the cells stimulated with LY + Met CM had an inhibition ratio similar to that of the control cells. **F**, **G** An in vivo study demonstrated that the tumorigenic ability of Cal27 cells was upregulated by LY CM but not by LY + Met CM. Tissue immunofluorescence staining (400×) showed that ALDH1A1 and CD44 levels were upregulated in tumors in the LY CM group but not in those in the LY + Met CM group. Bar: 100 μm. **H** Immunohistochemistry results (400×) for the HSC6 xenograft model showed that ALDH1A1 and CD44 levels were upregulated in tumors in the LY group but not in those in the LY + Met group. Bar: 100 μm. *P < 0.05 when compared with the control group; ^#^*P* < 0.05 when compared with the combined group; ns indicates no significant difference; one-way ANOVA. LY: LY2835219; Met: metformin; CM: conditioned medium.

Considering that an increase in cancer stemness is one of the most important pro-tumor effects of the SASP, we asked whether modulation of the SASP by the combination treatment could inhibit cancer stemness. In a sphere-forming assay, CM from cells treated with LY2835219 (LY CM) significantly enhanced the sphere-forming ability of cancer cells, while CM from the combination group (LY + Met CM) attenuated this effect (Fig. 4A). These observations were also confirmed with MCF7 cells (Supplementary Fig. S7). WB results showed that the expression of the cancer stem cell markers ALDH1A1, CD44, and Nanog was upregulated when cells were stimulated with LY CM, but this upregulation was diminished when cells were treated with LY + Met CM (Fig. 4B). This alteration was confirmed by an immunofluorescence assay (Fig. 4C) and flow cytometric analysis (Fig. 4D). Next, we tested whether increased stemness is associated with drug resistance and enhanced tumorigenesis. Cells pre-stimulated with CM from different groups were then treated with LY2835219. A CCK8 assay revealed that the cells pre-stimulated with LY CM had a significantly smaller inhibition ratio than the control cells (Fig. 4E). However, the cells pre-stimulated with LY + Met CM had an inhibition ratio similar to that of the control cells (Fig. 4E). To illuminate the tumorigenic ability of CM-treated cells in vivo, cells were pre-cultured with CM from different groups and then injected subcutaneously into mice. The results showed that the cells in the LY CM group displayed a stronger tumorigenic ability than those in the control group, while there was no significant difference between the LY + Met CM group and the control group (Fig. 4F, Supplementary Fig. S8). The formed tumors were then collected for immunofluorescence analysis. Overexpression of CD44 and ALDH1A1 was observed in the tumors of the LY CM group but not in the tumors of the LY + Met CM group (Fig. 4G). In addition, we detected the expression of stemness markers in tumors derived from the HSC6 xenograft model. We found that LY2835219 treatment increased the expression of CD44 and ALDH1A1 in vivo, but this effect was lost in the combination treatment group (Fig. 4H). Collectively, our results suggested that the combination of LY2835219 with metformin effectively blocked the SASP-induced stemness caused by the CDK4/6 inhibitor.

As the SASP has important effects on tumor immune infiltration and activity^37^, we also preliminarily explored the effects of LY2835219 with or without metformin on immune cells. IHC results showed that there were more infiltrated myeloid cells (CD11b+) and myeloid-derived suppressor cells (Gr1+) in the tumors of the LY group, while there was no significant difference between the LY + Met group and the control group (Supplementary Fig. S9). The infiltration of NK cells (NKp46+) was similar among the groups. Furthermore, an in vitro transwell assay revealed that Ctrl CM, LY CM and LY + Met CM produced comparable recruitment of immune cells; however, the cells recruited by LY CM included an elevated proportion of CD11b-expressing cells (Supplementary Fig. S10A-B). Moreover, the CD11b+ cells in the LY CM group exhibited higher expression of the immunosuppressive factor Arg1 than those in the other groups (Supplementary Fig. S10C).

### Blockade of the IL6-stat3 axis by metformin is associated with stemness inhibition

**Fig. 5:**
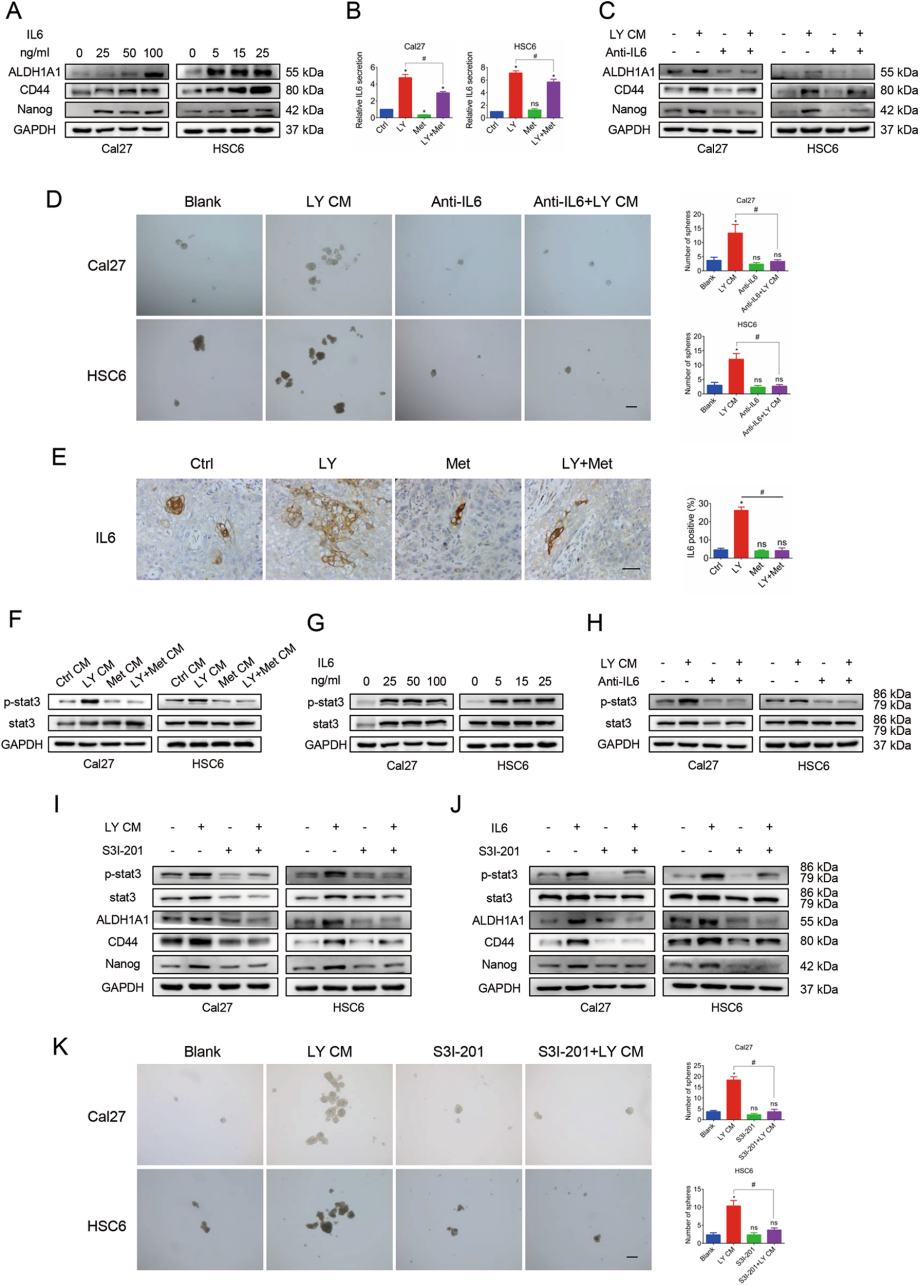
Metformin inhibited SASP-induced stemness by blocking the IL6-stat3 axis. **A** Western blot results showed that cells treated with IL6 had significantly increased expression of stemness markers. **B** ELISA results confirmed that the combined therapy decreased the IL6 secretion level, which was upregulated in the LY2835219 group. **C** Western blot results showed that an anti-IL6 neutralizing antibody effectively attenuated the increased expression of stemness markers induced by LY CM. **D** The anti-IL6 antibody abolished the SASP-enhanced sphere-forming ability (50×). Bar: 500 μm. **E** Immunohistochemistry results (400×) for the HSC6 xenograft model showed that IL6 expression was upregulated in tumors in the LY group but not in those in the LY + Met group. Bar: 100 μm. **F** Western blot results demonstrated that p-stat3 expression was upregulated by LY CM but not by LY + Met CM. **G** Cells stimulated with IL6 exhibited dramatic activation of the stat3 pathway. **H** The anti-IL6 antibody inhibited p-stat3, whose expression was upregulated by LY CM. **I**, **J** S3I-201 remarkably abrogated LY CM- or IL6-induced stat3 activation and stemness elevation. **K** A sphere-forming assay (50×) demonstrated that S3I-201 significantly abolished the LY CM-enhanced sphere-forming ability. Bar: 500 μm. *P < 0.05 when compared with the control group; ^#^*P* < 0.05 when compared with the combined group; ns indicates no significant difference; one-way ANOVA. LY: LY2835219; Met: metformin; CM: conditioned medium.

IL6 was overproduced in cells after treatment with LY2835219, and it was reported to be closely associated with cancer stemness. In our study, cells treated with IL6 exhibited significantly increased expression of stemness markers (Fig. 5A). Thus, we hypothesized that IL6 may be an important target to block SASP-induced stemness. ELISA results confirmed that the combination therapy decreased the secreted IL6 level, which was upregulated in the LY2835219 group (Fig. 5B). To further examine whether IL6 is responsible for SASP-induced stemness, a neutralizing antibody against IL6 was used to treat cells in the LY CM group. WB results showed that the anti-IL6 neutralizing antibody effectively attenuated the overexpression of stemness markers induced by the SASP (Fig. 5C). Consistently, the anti-IL6 antibody also abolished the SASP-enhanced sphere-forming ability (Fig. 5D). In tumors from the HSC6 xenograft model, we confirmed that IL6 was overexpressed in the LY2835219 group but not in the combination group (Fig. 5E). These findings suggest that IL6 plays a key role in SASP-induced stemness.

Next, we sought to determine which pathway participates in the enhancement of stemness mediated by IL6. WB results showed that the stat3 pathway was activated following LY CM treatment, but this effect was attenuated when cells were treated with LY + Met CM (Fig. 5F). Cells stimulated with IL6 consistently exhibited dramatic activation of the stat3 pathway (Fig. 5G). An anti-IL6 neutralizing antibody effectively inhibited the stat3 pathway, which was activated by LY CM (Fig. 5H). To further clarify the role of stat3 in SASP-induced stemness, cells were incubated with LY CM and the stat3 inhibitor S3I-201. The results showed that S3I-201 remarkably abrogated SASP-induced stat3 activation and stemness elevation (Fig. 5I). Similarly, S3I-201 attenuated the stat3 activation and stemness enhancement induced by IL6 (Fig. 5J). Moreover, a sphere-forming assay demonstrated that S3I-201 significantly abolished the SASP-enhanced sphere-forming ability (Fig. 5K). Taken together, our results reveal that metformin inhibits SASP-induced stemness through blockade of the IL6-stat3 axis.

**Fig. 6:**
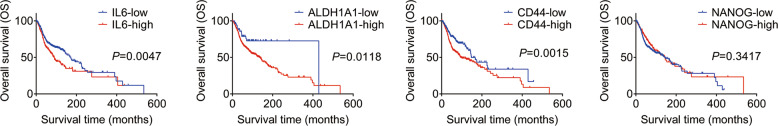
IL6 and cancer stemness markers were associated with poor survival in HNSCC. A survival analysis using the TCGA database showed that the overexpression of IL6 and the cancer stemness markers ALDH1A1 and CD44 was associated with poor overall survival in HNSCC. There was no significant association between Nanog and patient prognosis.

In addition, using the TCGA database, we found that overexpression of IL6, ALDH1A1, and CD44 was associated with relatively poor survival in HNSCC patients (Fig. 6). There was no significant association between Nanog expression and patient prognosis. These results indicate that IL6 and stemness markers may be therapeutic targets of metformin that can lead to an improved patient prognosis.

## Discussion

CDK4/6 inhibitors show promising antitumor activity in HNSCC. However, the SASP induced by CDK4/6 inhibitors is a double-edged sword in cancer treatment that can inhibit the growth of tumors but also promote tumor progression. Creating a strategy to minimize the deleterious effects while maintaining the beneficial impacts of the SASP is a serious challenge in the clinical application of CDK4/6 inhibitors. In this study, we found that metformin could act as a senostatic drug to modulate the profiles of the SASP elicited by a CDK4/6 inhibitor and thus enhance the anticancer effect on HNSCC.

As reported in two phase 1 clinical trials, a CDK4/6 inhibitor in combination with cetuximab was proven to be safe and resulted in an objective response in recurrent/metastatic HNSCC^38,39^. Furthermore, a phase 2 trial showed that the combination of a CDK4/6 inhibitor and cetuximab exhibited substantial antitumor activity in HNSCC, even in cetuximab-resistant patients, strongly suggesting that inhibition of CDK4/6 is a potential targeted therapeutic strategy in HNSCC^40^. However, failure to respond and response followed by progression were also observed in the study^40^. Another phase 2 trial reported that the combination of a CDK4/6 inhibitor and carboplatin exhibited insufficient antitumor activity in HNSCC^41^. These data collectively indicate that CDK4/6 inhibitors are promising in HNSCC, but further studies are warranted to explore the addition of other strategies to improve efficacy. For instance, CDK4/6 inhibitors in combination with immunotherapy (anti-PD-1 antibodies: NCT03655444, NCT04213404, and NCT04169074), a SHP2 inhibitor (TNO155: NCT04000529), or intensity-modulated radiation therapy (NCT03024489) are now being evaluated in clinical trials (clinicaltrials.gov).

TIS and the subsequent SASP are considered common effects elicited by CDK4/6 inhibitor treatment^9^. Considering their potential for tumor promotion, strategies to address TIS and the SASP may be the key to improving the anticancer efficacy of CDK4/6 inhibitors. Senotherapy was recently proposed to be an adjuvant strategy to address TIS in cancer treatment^23^. The two major types of senotherapies are senolytics and senostatics. Senolytic drugs, such as dasatinib, quercetin, ABT-263, and ABT-737, can effectively kill and eliminate senescent cells^42–44^. However, their uncertain anticancer effect^45^ and severe toxicity^46–48^ have hindered further clinical application of these compounds. Unlike senolytics, senostatics aim to interfere with TIS by targeting the SASP. The first studied senostatic agent was rapamycin, which nonselectively suppressed the SASP^49,50^. However, it is known that there are two biological effects of the SASP in the context of cancer treatment. Some molecules can drive the proliferation, survival, and metastasis of cancers, while others contribute to tumor suppression by maintaining growth arrest and enhancing immune clearance. Thus, nonselective suppression of SASP molecules may impair the anticancer efficacy to some extent. As reported, administration of rapamycin or BAY11-7082 can effectively suppress the SASP in cancer treatment; however, the beneficial impacts of the SASP, such as senescence maintenance and immune clearance, are then also eliminated, leading to tumor recurrence^51,52^. Therefore, another strategy of senostatics, SASP modulation, which refers to inhibiting pro-tumor SASP molecules while retaining the expression of antitumor SASP molecules, may be a more useful approach in cancer treatment. Toso et al. first proved the feasibility of this strategy. Suppression of the Jak2-stat3 pathway with maintained activation of the NF-κB pathway could reprogram the profiles of the SASP, thus enhancing the efficacy of senescence-inducing chemotherapy in prostate cancer^53^. The strategy of SASP modulation is promising; however, more experimental and clinical evidence is needed because of the context-dependent characteristics of the SASP. In this study, we found that metformin modulated the profiles of the SASP induced by a CDK4/6 inhibitor in HNSCC by suppressing the mTOR/stat3 pathway. The combination of metformin and the CDK4/6 inhibitor synergistically inhibited the growth of HNSCC in vitro and in vivo, suggesting that metformin can enhance anticancer efficacy as a senostatic drug.

Cancer stemness is known to contribute to tumor initiation, progression, and drug resistance. Upregulation of cancer stemness is one of the most important side effects caused by TIS and the SASP^54^. Addressing this increased cancer stemness is crucial in senescence-inducing chemotherapy. In this study, we found that LY CM indeed enhanced the in vitro sphere-forming and in vivo tumorigenic abilities of cancer cells, while LY + Met CM attenuated these effects. These data indicated that modulation of SASP profiles by metformin could block SASP-induced stemness in HNSCC. Furthermore, we proved that metformin exerted this effect by blocking the activation of the IL6-stat3 axis induced by a CDK4/6 inhibitor. The overexpression of IL6 and the cancer stemness markers CD44 and ALDH1A1 was associated with poor survival in HNSCC patients, suggesting that the metformin-mediated interference with the IL6-stat3 axis that inhibited SASP-induced stemness might improve the survival of HNSCC patients.

In addition, the SASP is reported to have dual effects on the antitumor immune response^37^. On the one hand, the SASP can suppress the antitumor immune response by recruiting immunosuppressive myeloid cells to the tumor site^55,56^. On the other hand, the SASP may stimulate the antitumor immune response by recruiting innate and adaptive immune cells (such as macrophages, neutrophils, NK cells, and T cells) to mediate senescent tumor cell clearance^57–59^. In our study, more infiltrated myeloid cells were observed in the LY group than in the other groups. The CDK4/6 inhibitor-induced SASP might generate an immunosuppressive microenvironment, which would weaken the anticancer efficacy of the inhibitor, while combination with metformin could attenuate this adverse effect. However, further study in immunocompetent mice is needed to clarify this effect.

**Fig. 7:**
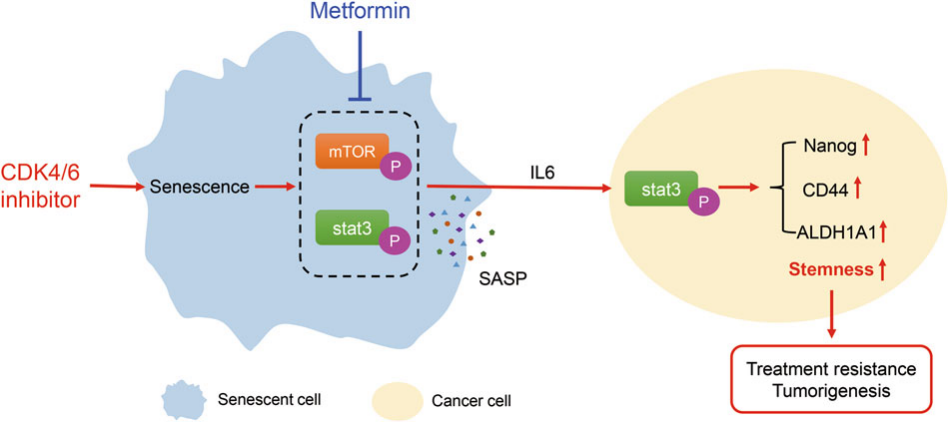
Schematic Diagram. Schematic showing metformin blocking CDK4/6 inhibitor-induced stemness by modulating the SASP.

Overall, our study revealed that monotherapy with a CDK4/6 inhibitor-induced senescence and the SASP in HNSCC. The main SASP factor, IL6, activated the stat3 pathway in neighboring cancer cells and thus increased cancer stemness, which led to treatment resistance and tumorigenesis. A combination with metformin suppressed the mTOR and stat3 pathways in senescent cancer cells to modulate the profiles of the therapy-induced SASP. As a result, the IL6-stat3 axis was blocked, and SASP-induced stemness was abrogated (Fig. 7). These results suggest that metformin can act as a senostatic drug to enhance the anticancer efficacy of CDK4/6 inhibitors by reprogramming the profiles of the SASP. These data provide new insights for the treatment of HNSCC and a new strategy for adjuvant tumor therapy.

## Supplementary information

Supplementary Figure Legends

Supplementary Table

Supplementary Figure S1

Supplementary Figure S2

Supplementary Figure S3

Supplementary Figure S4

Supplementary Figure S5

Supplementary Figure S6

Supplementary Figure S7

Supplementary Figure S8

Supplementary Figure S9

Supplementary Figure S10
