# Supplementary material for: Metformin as a senostatic drug enhances the anticancer efficacy of CDK4/6 inhibitor in head and neck squamous cell carcinoma
Source: Cell Death Dis. 2020 Oct 28;11(10):925. doi: 10.1038/s41419-020-03126-0 (PMC7595194; doi:10.1038/s41419-020-03126-0)
Supplement: Supplementary file 1 — Supplementary Figure Legends [file 41419_2020_3126_MOESM1_ESM.docx]

**Figure S1 The establishment of a PDX model.** Histopathological HE staining indicated that the histological characteristics of the PDX tumors after passaging were similar to those of the primary tumor. Bar: 200 μm. PDX: Patient-derived xenograft.

**Figure S2 The Cyclin D1-CDK4/6 pathway was overexpressed and associated with poor survival in HNSCC.** (A) The analysis results in the TCGA database showed that *CDK4*, *CDK6*, and *CCND1* were overexpressed in HNSCC. (B) Survival analysis revealed that patients with high expression of *CDK4*, *CDK6*, and *CCND1* exhibited poor overall survival. TCGA: The Cancer Genome Atlas.

**Figure S3 The combination of LY2835219 and metformin didn’t cause obvious change in mouse body weight and pathology of the liver and kidney.** (A) The body weight curves of the mice in the PDX model after treatment. (B) HE staining (200×) indicated that no obvious damage was observed in the liver and kidney tissues of the mice after treatment. Bar: 200 μm. PDX, Patient-derived xenograft; LY: LY2835219; Met: Metformin.

**Figure S4 The influence of LY2835219 and metformin on cell apoptosis.** Annexin V/PI staining results showed the cell apoptosis induced by LY2835219 with or without metformin. * indicates *P* < 0.05 when compared with the control group; ns indicates no significant difference; one-way ANOVA. LY: LY2835219; Met: Metformin.

**Figure S5 The influence of LY2835219 and metformin on cell senescence and the SASP in MCF7.** (A) SA-β-gal staining (100×) showed that remarkable senescent cells were elicited by the CDK4/6 inhibitor LY2835219 but not by metformin. Compared with LY2835219 monotherapy, the proportion of senescent cells unchanged after combined with metformin. Bar: 200 μm. (B) qRT-PCR results revealed that *IL6, IL8, MCP1, CXCL1, CXCL2*, and *CXCL3* were upregulated by LY2835219, while combination with metformin inhibited this upregulation. * indicates *P* < 0.05 when compared with the control group; # indicates *P* < 0.05 when compared with the combined group; ns indicates no significant difference; one-way ANOVA. LY: LY2835219; Met: Metformin.

**Figure S6 Metformin didn’t affect the antitumor SASP induced by LY2835219.** (A) qRT-PCR results showed that the SASP factors reported to be associated with antitumor effects, such as *IL1α, IL1β, TGFβ,* and *CCL5*, were not inhibited by metformin. (B-C) SA-β-gal staining (100×) revealed that LY CM, as well as LY+Met CM, could induce senescence in Cal27 cells. Bar: 400 μm. (D) A CCK8 assay showed that LY CM, as well as LY+Met CM, could inhibit the proliferation of Cal27 cells. The inhibitory effect of LY+Met CM was more pronounced. * indicates *P* < 0.05 when compared with the control group; # indicates *P* < 0.05 when compared with the combined group; ns indicates no significant difference; one-way ANOVA. LY: LY2835219; Met: Metformin; CM: Conditioned medium.

**Figure S7 Metformin blocked the SASP-induced stemness which was caused by LY2835219 in MCF7.** A sphere-forming assay (50×) showed that LY CM significantly enhanced the sphere-forming ability of MCF7 cells, while LY+Met CM attenuated this effect. Bar: 500 μm. * indicates *P* < 0.05 when compared with the control group; # indicates *P* < 0.05 when compared with the combined group; ns indicates no significant difference; one-way ANOVA. LY: LY2835219; Met: Metformin; CM: Conditioned medium.

**Figure S8 Combination of LY2835219 with metformin blocked the SASP-enhanced tumorigenic ability *in vivo*.** Cal27 cells were incubated with CM from different groups for 96 h. Then, 2*10^5^ cells in different groups were injected subcutaneously into the right armpit of nude mice (n=8) in a blinded fashion. After 1 month, the mice were sacrificed and the tumors formed were recorded. LY: LY2835219; Met: Metformin; CM: Conditioned medium.

**Figure S9 The influence of LY2835219 and metformin on immune cell infiltration.** IHC staining was performed to detect the infiltration of myeloid cells (CD11b+), myeloid-derived suppressor cells (Gr1+), and NK cells (NKp46+) into tumors in the HSC6 xenograft model. Bar: 200 μm. * indicates *P* < 0.05, ns indicates no significant difference; one-way ANOVA. LY: LY2835219; Met: Metformin.

**Figure S10 The *in vitro* influences of conditioned medium on immune cell recruitment and activity.** (A) A transwell migration assay was conducted to illustrate the recruitment of immune cells by conditioned medium (CM) from different groups. Human peripheral blood mononuclear cells (10^6^ cells per well) were placed in the upper chamber, while CM from different groups was placed in the lower chamber. The cells that migrated to the lower chamber after 24 h were counted and compared. (B) Cells that migrated to the lower chamber were tested for the expression of CD11b by flow cytometry. (C) CD11b+ cells were further tested for the expression of the immunosuppressive factor Arg1 by flow cytometry. * indicates *P* < 0.05, ns indicates no significant difference; one-way ANOVA. LY: LY2835219; Met: Metformin; CM: Conditioned medium.
