# Supplementary material for: Metformin as a senostatic drug enhances the anticancer efficacy of CDK4/6 inhibitor in head and neck squamous cell carcinoma
Source: Cell Death Dis. 2020 Oct 28;11(10):925. doi: 10.1038/s41419-020-03126-0 (PMC7595194; doi:10.1038/s41419-020-03126-0)
Supplement: Supplementary file 2 — Supplementary Table [file 41419_2020_3126_MOESM2_ESM.docx]

**Supplementary Table**

**Supplementary Table 1**

| Genes | Primers | |
| --- | --- | --- |
| *IL6* | Forward Primer: 5’- ACTCACCTCTTCAGAACGAATTG -3’  Reverse Primer: 5’- CCATCTTTGGAAGGTTCAGGTTG -3’ |  |
| *IL8* | Forward Primer: 5’- TGTGAAGGTGCAGTTTTGCCA -3’  Reverse Primer: 5’- ATTTCTGTGTTGGCGCAGTG -3’ | |
| *MCP1* | Forward Primer: 5’- CTGTGCCTGCTGCTCATAG -3’  Reverse Primer: 5’- CTTGCTGCTGGTGATTCTTCT -3’ | |
| *CXCL1* | Forward Primer: 5’- GCTGAACAGTGACAAATCCAAC -3’  Reverse Primer: 5’- CTTCAGGAACAGCCACCAGT -3’ | |
| *CXCL2* | Forward Primer: 5’- CCCATGGTTAAGAAAATCATCG -3’  Reverse Primer: 5’- CTTCAGGAACAGCCACCAAT -3’ | |
| *CXCL3* | Forward Primer: 5’- AAGTGTGAATGTAAGGTCCCC -3’  Reverse Primer: 5’- GTGCTCCCCTTGTTCAGTATC -3’ | |
| *IL1α* | Forward Primer: 5’- TGGTAGTAGCAACCAACGGGA -3’  Reverse Primer: 5’- ACTTTGATTGAGGGCGTCATTC -3’ | |
| *IL1β* | Forward Primer: 5’- TGAAGCAGCCATGGCAGAAG -3’  Reverse Primer: 5’- GGTCGGAGATTCGTAGCTGGA -3’ | |
| *TGF-β* | Forward Primer: 5’- CTAATGGTGGAAACCCACAACG -3’  Reverse Primer: 5’- TATCGCCAGGAATTGTTGCTG -3’ | |
| *CCL5* | Forward Primer: 5’- AGTCGTCTTTGTCACCCGAAAG -3’  Reverse Primer: 5’- TCCCGAACCCATTTCTTCTCT -3’ | |
| *GAPDH* | Forward Primer: 5’- TGACAACAGCCTCAAGAT -3’  Reverse Primer: 5’- GAGTCCTTCCACGATACC -3’ | |
