# Supplementary figures and images for: Metformin as a senostatic drug enhances the anticancer efficacy of CDK4/6 inhibitor in head and neck squamous cell carcinoma
Source: Cell Death Dis. 2020 Oct 28;11(10):925. doi: 10.1038/s41419-020-03126-0 (PMC7595194; doi:10.1038/s41419-020-03126-0)

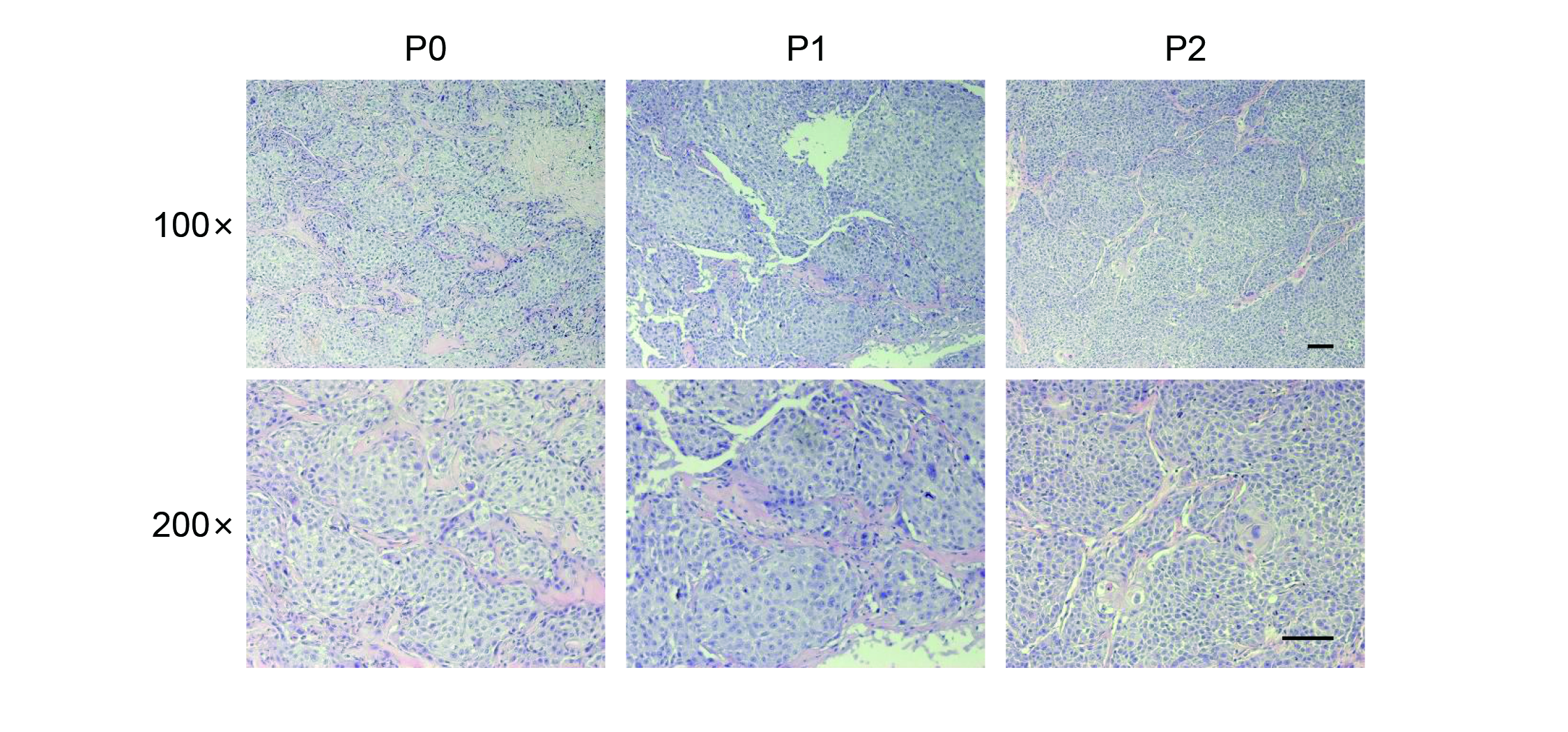

Supplement: Supplementary file 3 — Supplementary Figure S1 [file 41419_2020_3126_MOESM3_ESM.tif]

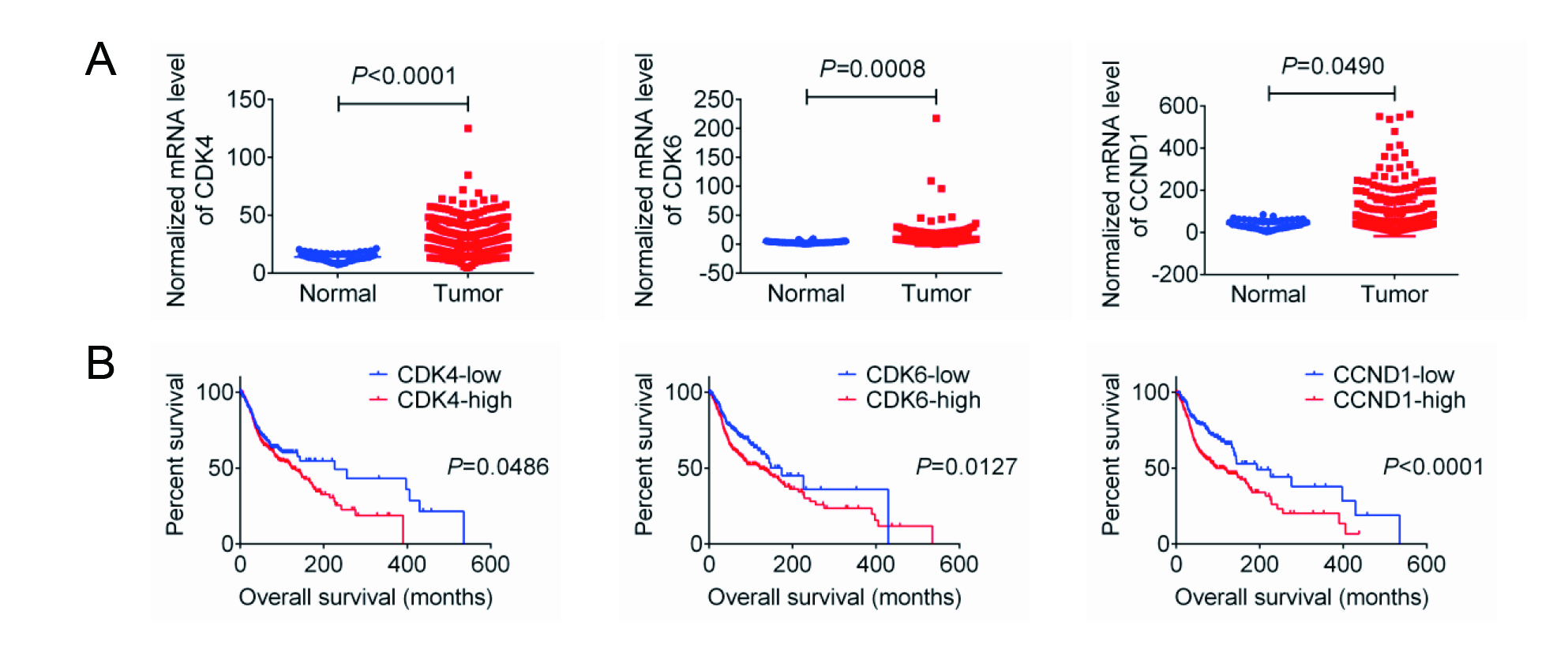

Supplement: Supplementary file 4 — Supplementary Figure S2 [file 41419_2020_3126_MOESM4_ESM.tif]

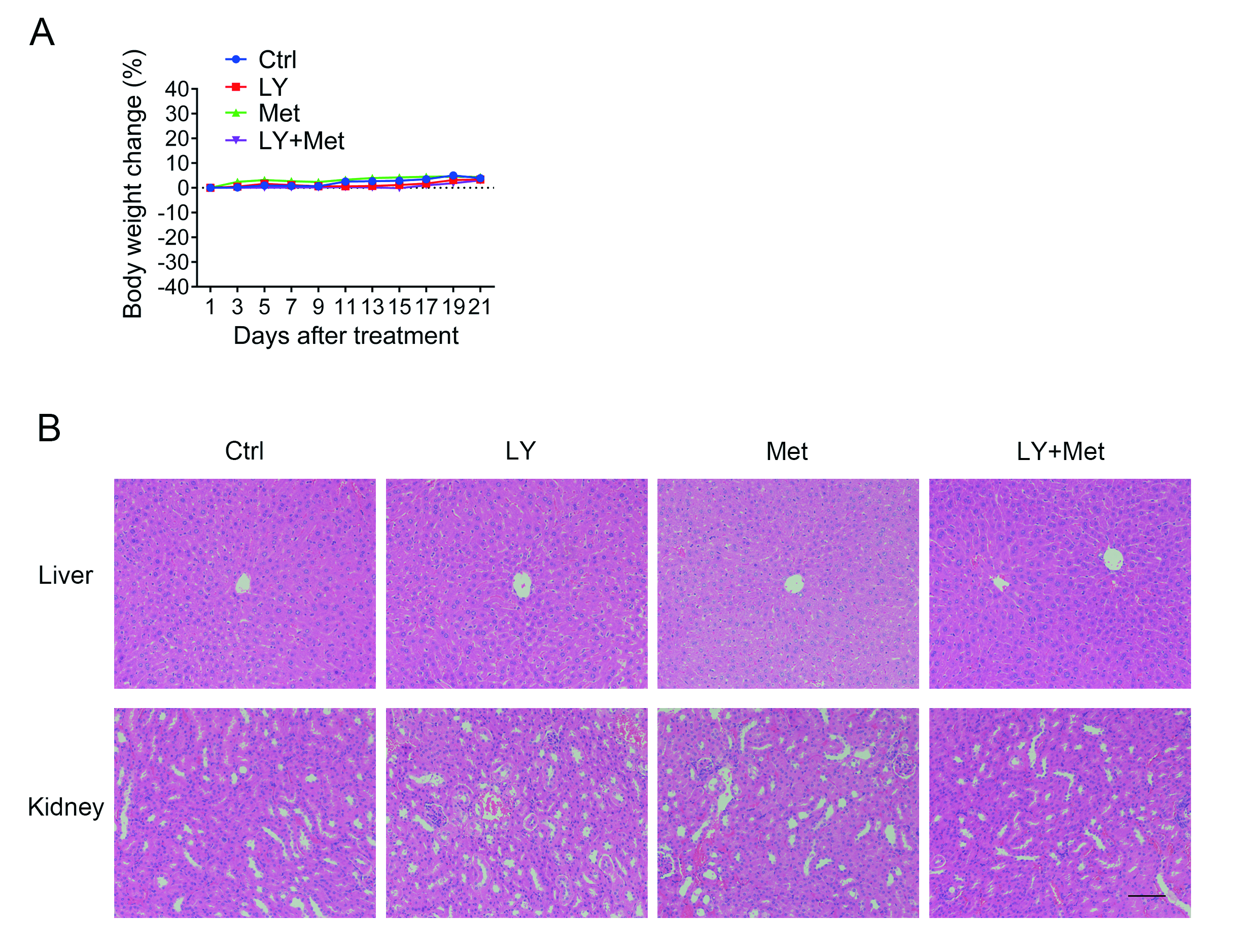

Supplement: Supplementary file 5 — Supplementary Figure S3 [file 41419_2020_3126_MOESM5_ESM.tif]

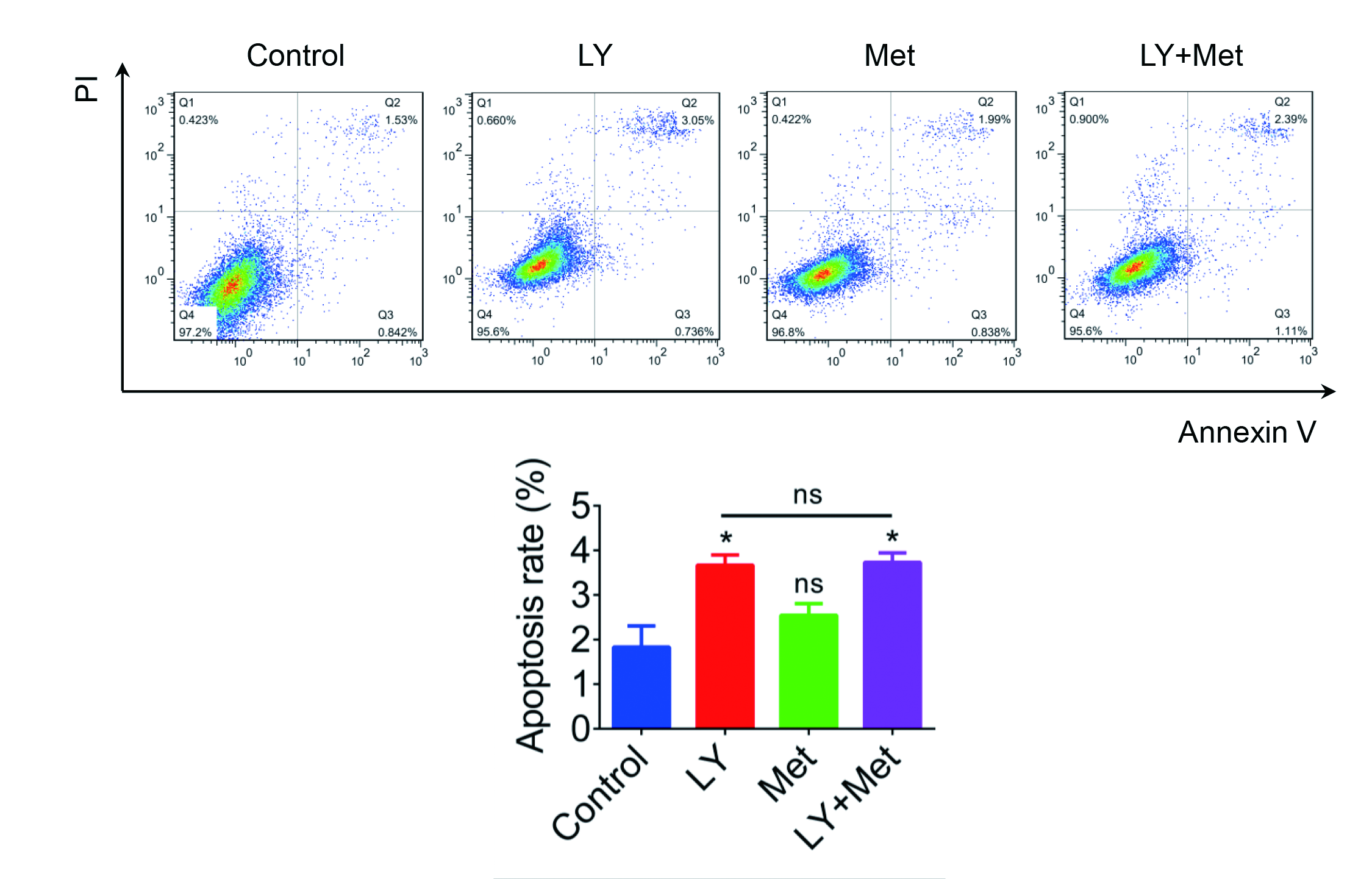

Supplement: Supplementary file 6 — Supplementary Figure S4 [file 41419_2020_3126_MOESM6_ESM.tif]

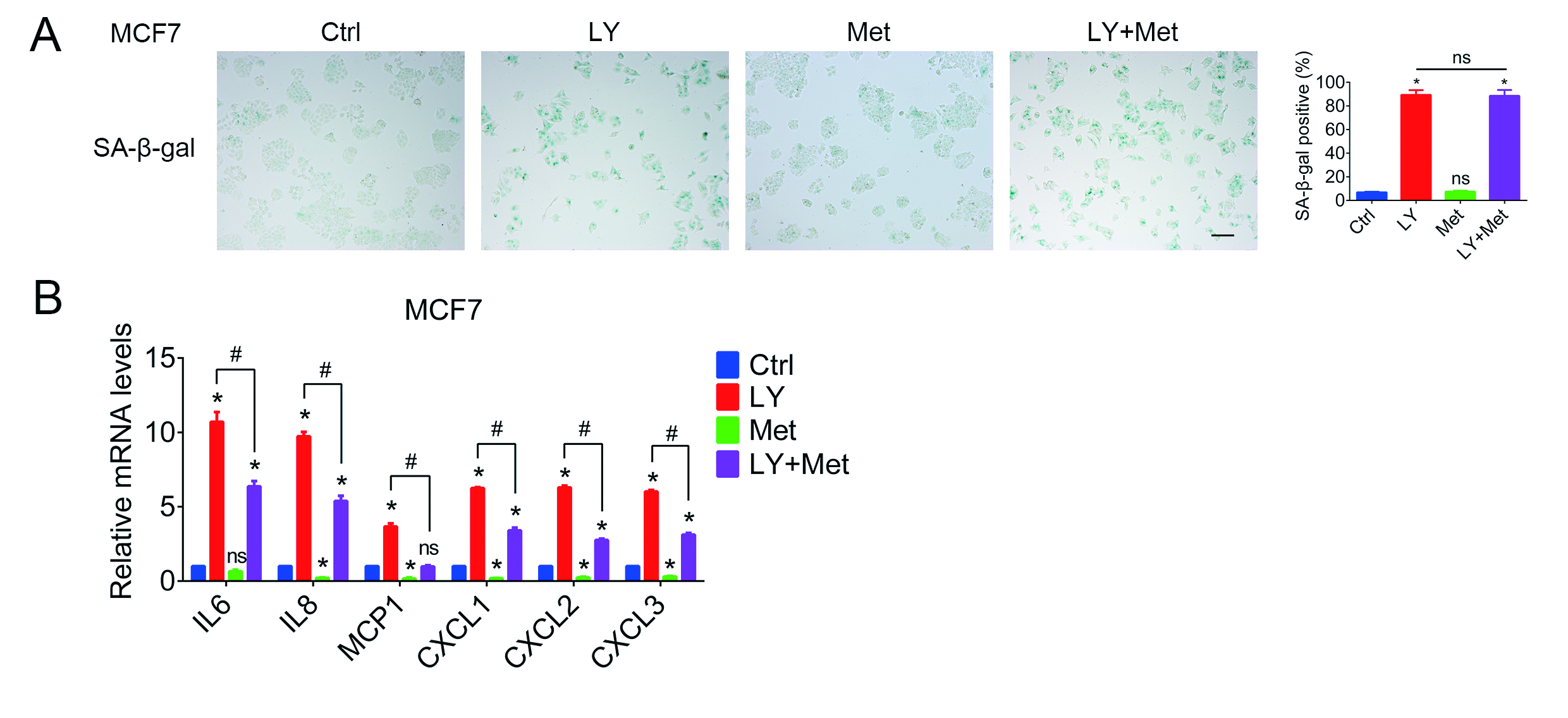

Supplement: Supplementary file 7 — Supplementary Figure S5 [file 41419_2020_3126_MOESM7_ESM.tif]

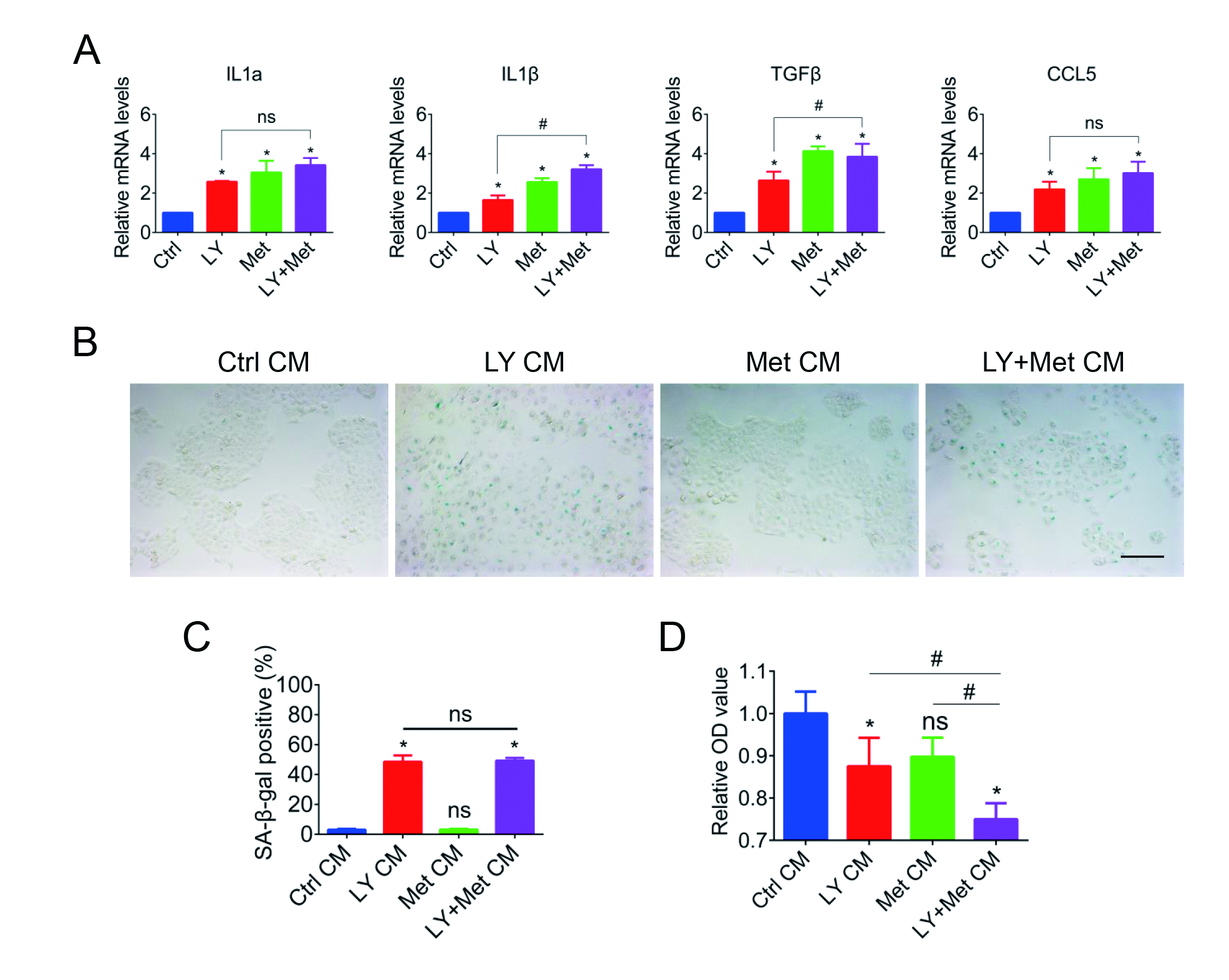

Supplement: Supplementary file 8 — Supplementary Figure S6 [file 41419_2020_3126_MOESM8_ESM.tif]

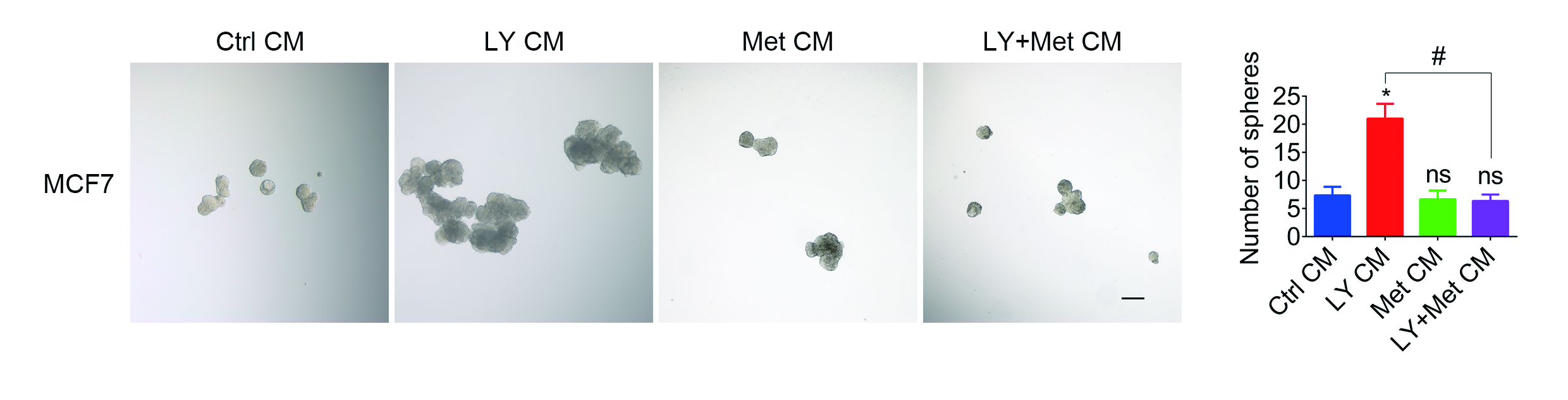

Supplement: Supplementary file 9 — Supplementary Figure S7 [file 41419_2020_3126_MOESM9_ESM.tif]

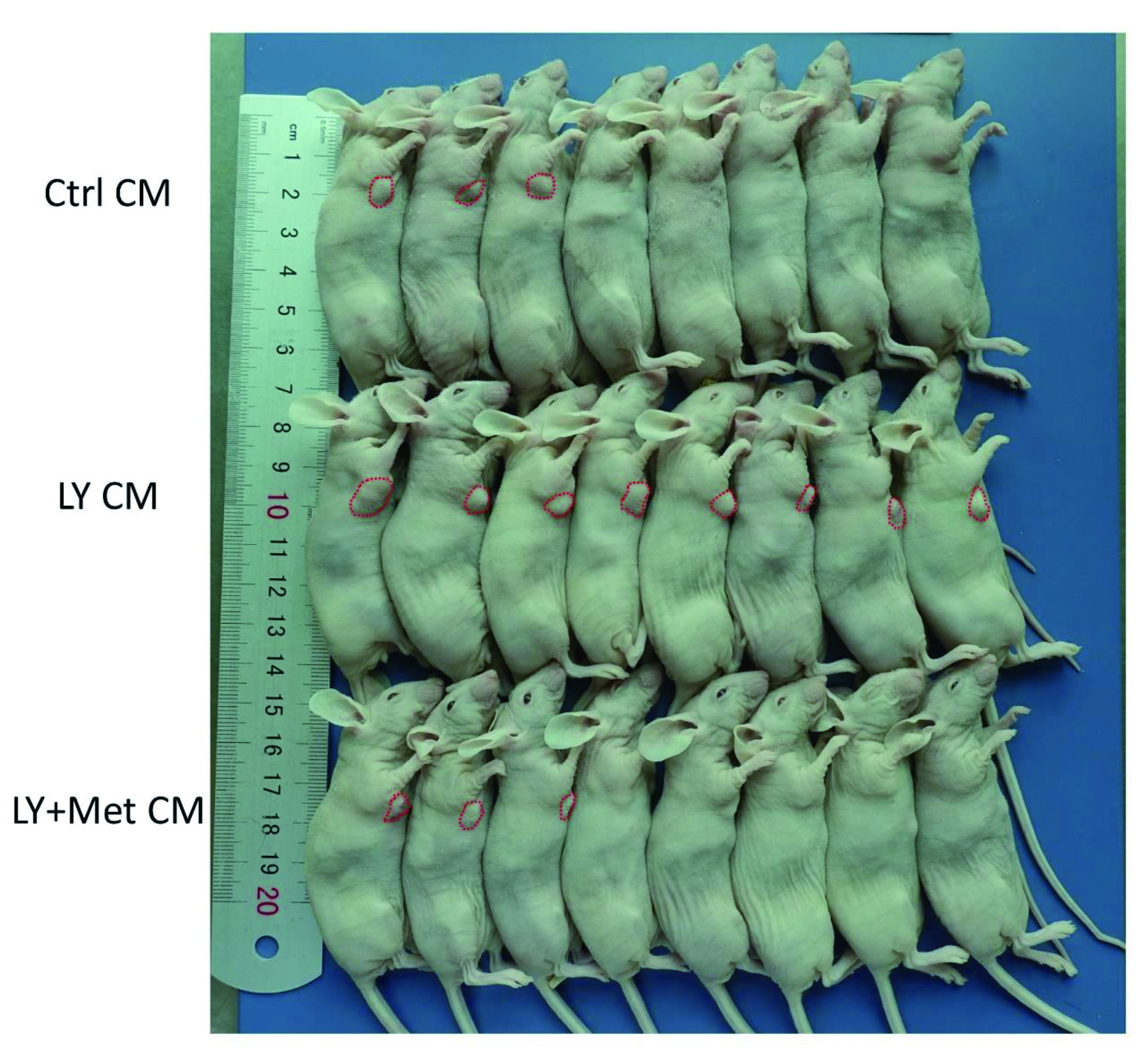

Supplement: Supplementary file 10 — Supplementary Figure S8 [file 41419_2020_3126_MOESM10_ESM.tif]

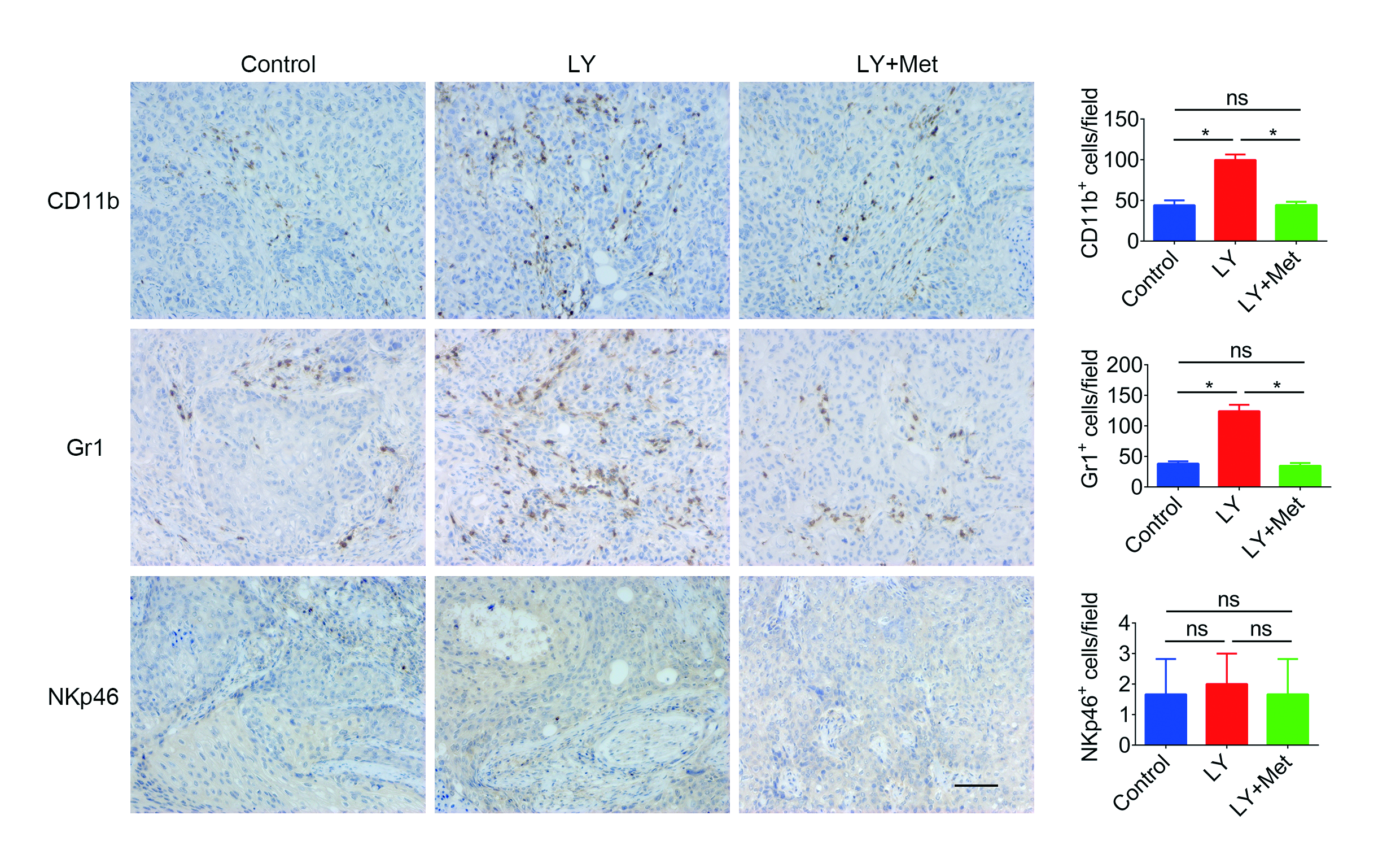

Supplement: Supplementary file 11 — Supplementary Figure S9 [file 41419_2020_3126_MOESM11_ESM.tif]

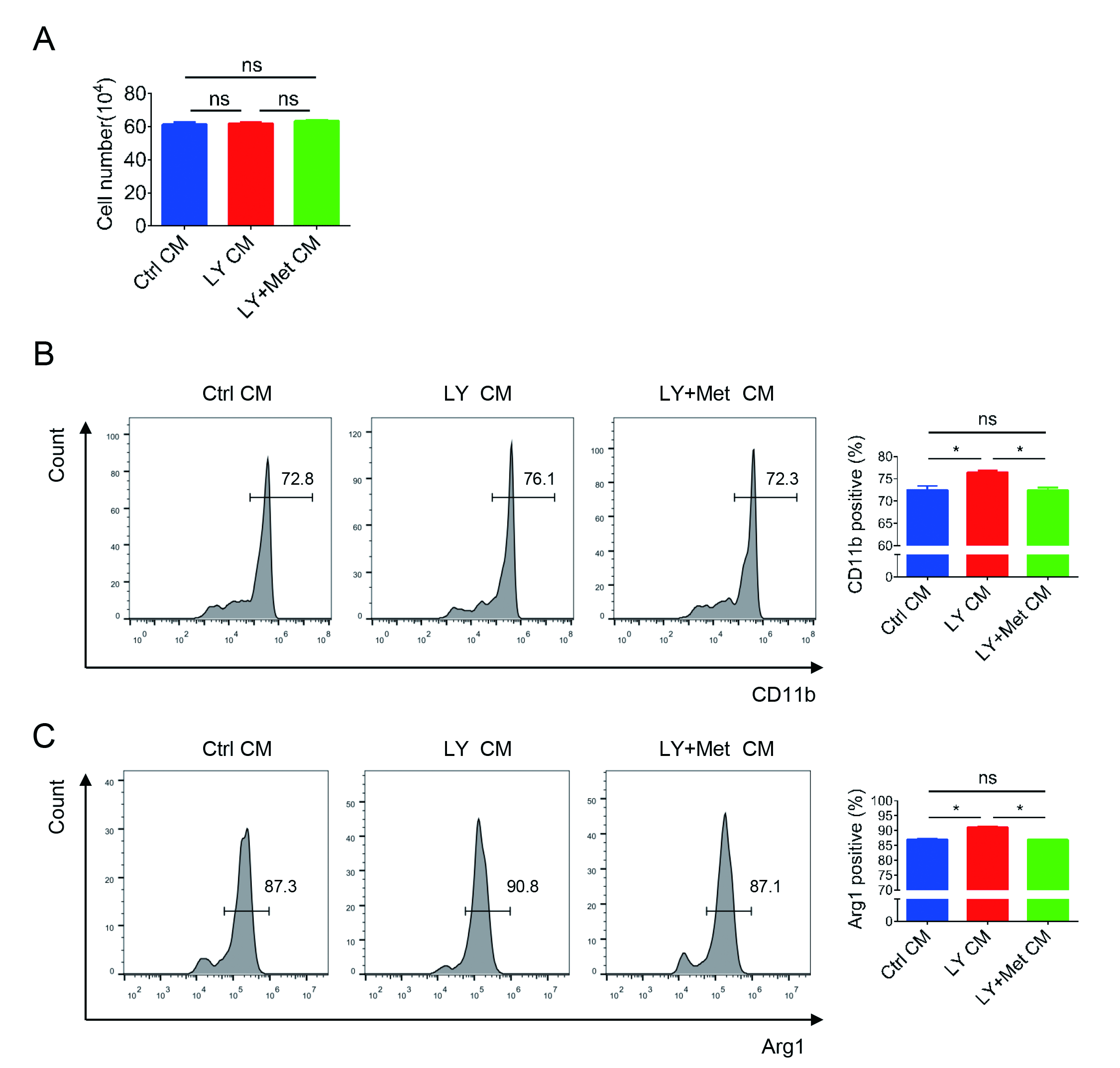

Supplement: Supplementary file 12 — Supplementary Figure S10 [file 41419_2020_3126_MOESM12_ESM.tif]
